# Structure of the *Chimalliviridae* bacteriophage Goslar reveals host recognition and infection machinery

**DOI:** 10.1126/sciadv.aei4524

**Published:** 2026-11-06

**Authors:** Dwaipayan Basu, Yajie Gu, Ying-Xing Li, Taylor Forman, Majid Ghassemian, Ryan K. Min, Joe Pogliano, Kevin D. Corbett

**Affiliations:** ^1^ Department of Biochemistry and Molecular Biophysics, University of California, San Diego, La Jolla, CA 92093, USA.; ^2^ Department of Cellular & Molecular Medicine, University of California, San Diego, La Jolla, CA 92093, USA.; ^3^ Department of Molecular Biology, University of California, San Diego, La Jolla, CA 92093, USA.

## Abstract

Large-genome bacteriophages (phages) of the *Chimalliviridae* family have a distinctive life cycle, building compartments inside infected bacterial cells that broadly protect their genomes from host defenses. Here, we use cryo–electron microscopy to determine a structure of the *Escherichia coli*–infecting *Chimalliviridae* phage Goslar. We combine manual modeling of 25 structural proteins with AlphaFold structure predictions of an additional three proteins to generate a composite model with 2888 protein chains from 28 different structural proteins, totaling more than 1.4 million amino acid residues. Our structure reveals the architecture of the Goslar capsid, portal, tail, and baseplate, highlighting structural similarities and differences with other tailed phages. Combining our structural data with quantitative mass spectrometry of Goslar virions, we identify several high-copy virion-associated proteins that likely play key roles when injected into host cells upon infection. We also identify a *Chimalliviridae*-specific family of proteins that form long, flexible filaments anchored at the phage baseplate and which incorporate carbohydrate-binding domains, suggesting a key role in host recognition.

## INTRODUCTION

Bacteriophages (phages) are the most abundant organisms on Earth, outnumbering their bacterial prey by an order of magnitude (*1*, *2*). Phages show huge diversity in their genome sizes—ranging from 3 to 5 kb for *Microvidiae* (*3*) to more than 800 kb for so-called jumbo phages (*4*–*6*)—as well as in their physical morphology and overall life cycle. Historically, jumbo phages (classified as those with genomes more than 200 kb) have been understudied because typical methods did not allow for their isolation. Recent studies using deep-sequencing data from environmental samples (*4*) and/or new isolation methods (*7*, *8*), however, have revealed that jumbo phages are abundant and highly diverse (*9*).

One family of jumbo phages, termed *Chimalliviridae*, has a distinctive life cycle that enables it to evade DNA-targeting host defenses like CRISPR-Cas nucleases and restriction endonucleases (*10*–*15*). Upon initial infection, *Chimalliviridae* phages (here termed chimalliviruses) inject their genome into a membrane-enclosed vesicle termed the EPI (early phage infection) vesicle (*12*, *13*, *16*). A suite of phage proteins is injected into the host cell alongside the genome, including a multisubunit RNA polymerase (the virion RNA polymerase or vRNAP) (*17*–*19*), which transcribes early genes from within the EPI vesicle (*12*, *13*, *16*). One early-expressed protein is called chimallin (“shield”), and this protein self-assembles into a closed compartment termed the phage nucleus, into which the phage genome is transferred from the EPI vesicle (*11*, *20*, *21*). Inside the phage nucleus, the genome is replicated and late genes are transcribed by a second phage-encoded RNA polymerase (the non-vRNAP or nvRNAP) (*11*, *13*, *19*, *22*–*25*). Capsids are assembled in the host cytoplasm and then migrate to the phage nucleus, where they dock onto the chimallin shell for genome packaging (*11*, *20*, *26*). Capsids and tails are then assembled within a subcellular space termed the bouquet, followed by host-cell lysis (*26*–*28*).

Many questions remain regarding the complex life cycle of chimalliviruses. Notably, the segregation of the EPI vesicle and phage nucleus from the host cytoplasm means that transcribed mRNAs must be transported out of these compartments for translation by host ribosomes (*12*, *29*), and both phage- and host-encoded proteins are transported into the phage nucleus (*30*, *31*). Further, while low-resolution structural studies of intact chimalliviruses have been performed (*32*), their detailed architecture is not well understood, meaning that host recognition and infection mechanisms of this phage family remain mostly unknown. Last, the full complement of proteins injected alongside the chimallivirus genome upon initial infection is not known, leaving open the questions of how early genes are transcribed and how their mRNAs are transported out of the EPI vesicle.

Likely because of their broad resistance to the most abundant host defense pathways, combined with their diverse host-recognition mechanisms, many chimalliviruses show extremely broad host range and hold promise as antibacterial therapeutics (*8*, *33*). Addressing how these viruses recognize their host and navigate early-infection events could enable rational selection or engineering of these phages for therapies targeted to particular patient isolates.

Here, we use cryo–electron microscopy (cryo-EM) to determine a near-complete structure of one chimallivirus, the *Escherichia coli*–infecting phage Goslar, and use mass spectrometry to comprehensively define its protein payload. Our final molecular model comprises 2888 individual chains from 28 distinct proteins. Our structure, along with a recent structure of the *Pseudomonas*-infecting phage PhiKZ (*34*) defines the detailed architecture of chimalliviruses, revealing broad similarity to other tailed viruses (*Caudoviricetes*) with several distinctive features. Our mass spectrometry survey of virion-associated proteins reveals that each Goslar virion carries hundreds of copies of several structurally similar proteins thought to comprise an internal capsid structure called the “inner body” and play key roles upon injection into the host cell. Further, we identify a chimallivirus-specific protein family characterized by the DUF7941 domain; these proteins likely mediate host recognition by self-assembling into extended flexible filaments radiating up to several micrometers from the phage baseplate.

## RESULTS

### Cryo-EM structure determination of phage Goslar

To determine the overall structure of phage Goslar, we prepared high-titer phage lysates [∼10^9^ plaque-forming units (PFU)/ml] by infecting lawns of *E. coli* MC1000 cells on agar plates, collecting the resulting phage in a stabilizing buffer and then concentrating using centrifugal ultrafiltration. Further purification by polyethylene glycol (PEG) precipitation and CsCl gradient centrifugation did not result in higher particle density on cryo-EM grids, so we used samples before these steps for structure determination, and the more highly purified samples for mass spectrometry analysis (see below). We prepared cryo-EM samples on graphene-coated lacey carbon grids and initially collected a 4224-micrograph dataset on a Titan Krios G3 with Gatan K3 camera in super-resolution mode, with a super-resolution pixel size of 1.3 Å per pixel (dataset 1). From this dataset, we manually picked 3033 intact Goslar phage particles and calculated an ∼19-Å resolution asymmetric (C1) map of the full Goslar virion in cryoSPARC (fig. S1 and data S1) (*35*). In this map, the capsid, portal/neck, tail, and baseplate are clearly visible (Fig. 1A).

**Fig. 1.**
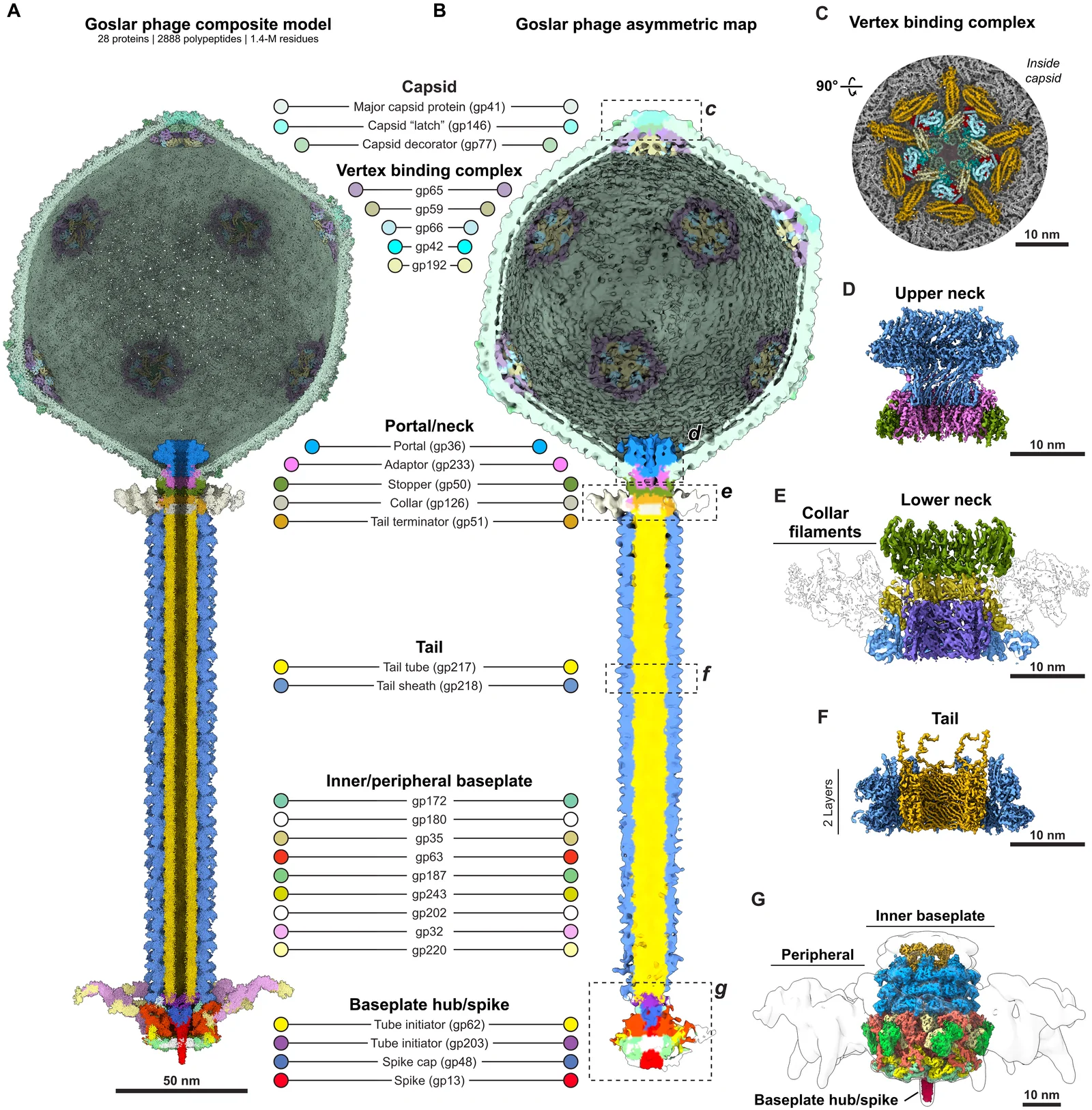
Overview of cryo-EM structure determination of the Goslar virion. (**A**) Composite model of the Goslar virion, comprising 2888 individual polypeptide chains from 28 different proteins (see table S1 for copy number of each protein). Proteins are colored according to the key. (**B**) Asymmetric map (∼19-Å resolution) of the Goslar virion, colored by protein as in (A). Dotted boxes indicate the positions of detail maps in (C) through (G). (**C**) A 3.6-Å resolution map of the capsid C5 penton vertex, viewed from inside the capsid with the VBC colored as indicated in (A) and (B). See Fig. 2 for further details of capsid structure and Fig. 3 for further details of the VBC. (**D**) A 3.8-Å resolution map of the C12-symmetric portal and upper neck, with portal protein (gp36) colored blue, adaptor protein (gp233) pink, and the C12-symmetric N-terminal domains of the stopper protein (gp50). (**E**) A 4.2-Å resolution map of the C6-symmetric lower neck. The C6-symmetric C-terminal domains of the stopper protein (gp50) are shown in green, with the tail terminator (gp51) in yellow, the tail tube (gp217) in purple, and the tail sheath (gp218) in blue. The collar and collar filaments (gp126) are shown as a transparent outline. See Fig. 4 for further details of the portal/neck and collar. (**F**) A 3.4-Å resolution map of the tail (two layers shown), with tail tube protein (gp217) in yellow and tail sheath (gp218) in blue. See Fig. 5 for further details of the tail. (**G**) A 3.9-Å resolution map of the baseplate, with proteins colored as in (A) and (B). The peripheral baseplate is shown as a transparent Gaussian-filtered map. See Fig. 6 for further details of the baseplate.

For high-resolution analysis, we separately collected two datasets totaling 13,518 micrographs on the same Titan Krios G3 with Gatan K3 camera in counting mode, with a pixel size of 1.4 Å per pixel (datasets 2 and 3). The smaller field of view in these micrographs resulted in fewer complete virions being visible, but the resulting reconstructions of individual substructures were higher-quality than those calculated from dataset 1. Using cryoSPARC (*35*), we pursued a multipronged strategy for structure determination of intact Goslar virions that leveraged the high symmetry of each substructure (see Materials and Methods, figs. S2 to S6, and data S2). In all, we generated separate cryo-EM maps for the full capsid [icosahedral (I1) symmetry, 6.3-Å resolution; figs. S2 and S3], empty capsid (I1 symmetry, 5.2-Å resolution; fig. S4), capsid vertex (C5 “penton,” 3.6-Å resolution; figs. S2 and S3), capsid hexamer (C3 “hexon,” 3.6-Å resolution; figs. S2 and S3), portal and upper neck (C12, 3.8-Å resolution; figs. S2 and S3), lower neck (C6, 4.2-Å resolution; figs. S2 and S3), tail (C6: intact tail, 3.4-Å resolution; tail tube, 3.2-Å resolution; tail sheath, 3.4-Å resolution; fig. S5), baseplate hub and wedges (C6, 3.9 Å resolution; fig. S6), and peripheral baseplate (7.9-Å resolution; fig. S6) (Fig. 1).

To build atomic models into each high-resolution map, we leveraged a combination of sequence identity to known phage structural proteins, sequence fragments assigned by ModelAngelo (*36*), three-dimensional (3D) models and predicted protein-protein interactions from AlphaFold 3 (*37*), and mass spectrometry of purified Goslar virions (see below) to identify and build models for each protein visible in the capsid, portal, neck, tail, and baseplate maps. In all, we built and refined atomic models for 2870 separate chains from 25 structural proteins (see fig. S7 for models and corresponding cryo-EM density). Our final composite model, which includes docked AlphaFold models for three additional structural proteins (18 separate chains), comprises 2888 protein chains from 28 different structural proteins and totals more than 1.4 million amino acid residues (Fig. 1B).

A primary motivation for our structure determination effort was to visualize the so-called inner body, a large structure previously observed in low-resolution cryo-EM studies of the *Pseudomonas*-infecting chimallivirus PhiKZ that forms a pillar-like assembly spanning the inside of the filled capsid, positioned diagonally with respect to the portal/tail axis (*38*, *39*). The inner body has been thought to comprise a set of structurally related proteins that are abundant in the virion (*40*–*42*), but this structure was not resolved in a recent capsid structure of PhiKZ potentially because of the use of icosahedral symmetry averaging in that work (*41*). A more recent study that included an ∼10-Å resolution asymmetric cryo-EM structure of the intact PhiKZ virion also did not observe density for the inner body (*34*). We did not observe density for the inner body in our primary micrographs of Goslar (fig. S1A), or in our asymmetric (C1) map of the Goslar virion (Fig. 1B), in which particles were unambiguously aligned with respect to both the capsid and portal/neck/tail substructures. To account for potential ambiguous alignment of the inner body in different particles, we performed C5 symmetry expansion of capsid particle images along the portal vertex and performed 3D classification (not shown); this effort also did not reveal density for the inner body. The lack of density for the inner body, combined with our inability to observe this structure in primary micrographs, suggests either that Goslar does not have a highly ordered inner body or that our imaging parameters did not allow for visualization of this structure. The Goslar capsid does contain several high-copy virion-associated proteins homologous to proteins from PhiKZ that have been thought to comprise the inner body; these are discussed more below.

### Capsid architecture

The Goslar capsid has a diameter of ∼147 nm and adopts an overall icosahedral (I1) symmetry (Fig. 2A), with the exception that at 1 of the 12 vertices, a pentamer of the major capsid protein (MCP; gp41) is replaced by the portal (described below). Each of the 60 asymmetric units (Fig. 2B) is composed of 27 copies of the MCP [triangulation number (T) = 27, h = 3, k = 3]. The total MCP copy number in the capsid is therefore 1615 [27 × 60 (I1 symmetry) = 1620 – 5 (portal vertex) = 1615]. Each asymmetric unit contains four complete MCP hexamers (hereafter referred to as hexons), one-third of a fifth hexon, and one-fifth of an MCP pentamer (hereafter referred to as a penton) (Fig. 2C). Our final models of the MCP in the penton and hexon contain 576 and 583 of the total 740 residues, respectively. No density for the N-terminal 157 residues of the MCP were observed in either structure, suggesting that the Goslar MCP is subject to proteolytic processing like the MCP of PhiKZ (*40*, *41*). Each asymmetric unit also contains one copy each of two proteins that decorate the outer capsid surface: the capsid “latch” protein gp146 binds and stabilizes the MCP penton, and the capsid decorator protein gp77 is a small globular protein bound to the outer surface between two adjacent MCP protomers (Fig. 2C). We clearly observe one copy of gp77 per asymmetric unit bound to hexon 1, but at least two additional copies per asymmetric unit are also visible at contour levels too low to build (not shown). Last, the asymmetric unit contains five additional proteins (gp42, gp59, gp65, gp66, and gp192) that form a “vertex-binding complex” (VBC) on the inner surface of the capsid (Fig. 2C; described in more detail next section).

**Fig. 2.**
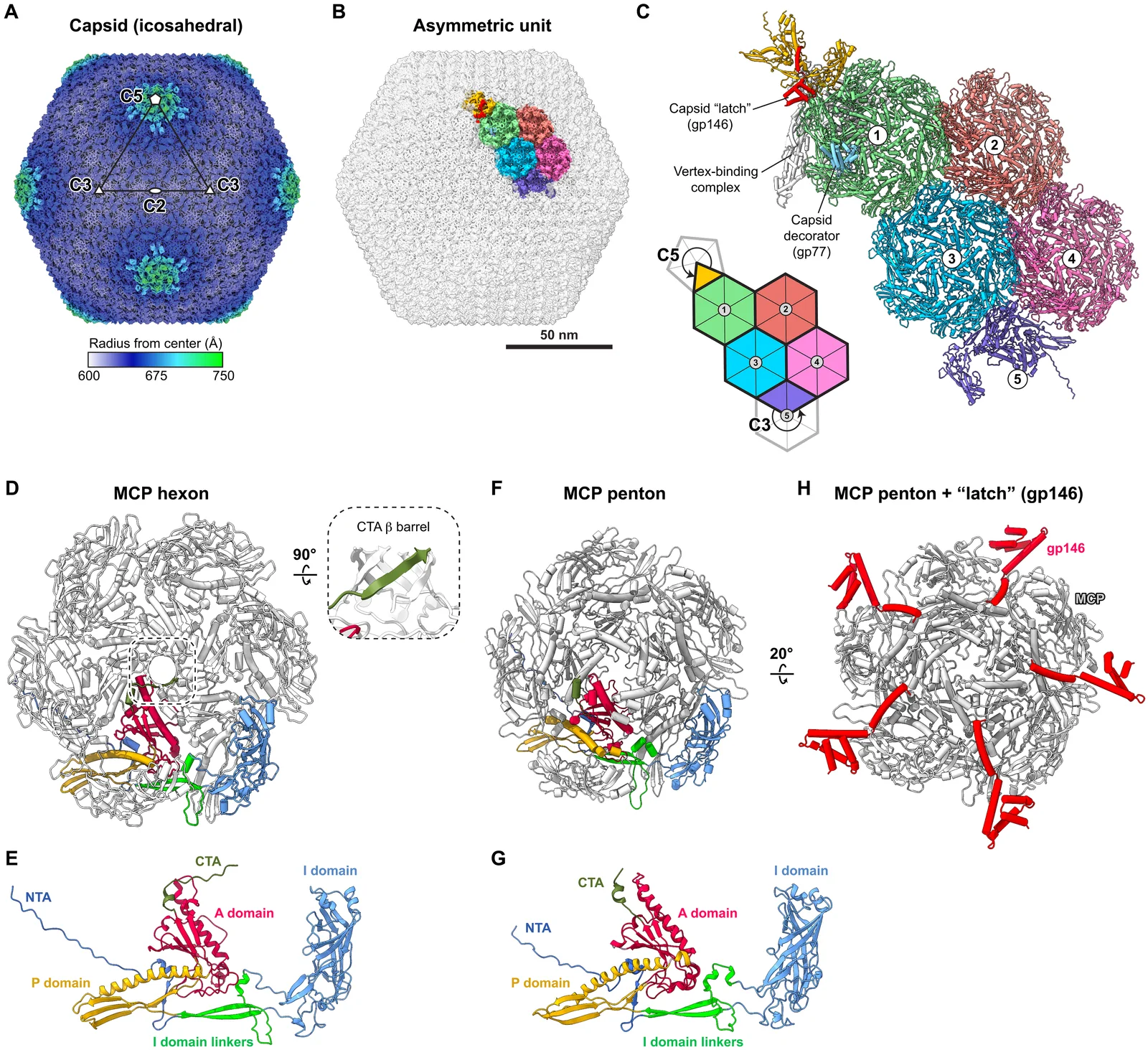
Goslar capsid architecture. (**A**) A 6.3-Å resolution icosahedrally averaged map of the Goslar capsid, colored by radius from center. The twofold (C2), threefold (C3), and fivefold (C5) symmetry axes are labeled. (**B**) Icosahedrally average map of the Goslar capsid with a single asymmetric unit colored. (**C**) Detail view of the Goslar capsid asymmetric unit, with inset diagram. The numbers 1 to 5 designate MCP hexons (hexon 5 is at a C3 symmetry axis). In addition to MCP, the capsid latch protein (gp146) is shown in red; the capsid decorator protein (gp77) is shown in light blue bound to hexon 1, and the VBC is shown in white. (**D**) Cartoon view of an MCP hexon, with one MCP protomer colored by domain as in (E). Inset: rotated view of the six-stranded β barrel formed by the CTA at the center of the hexon. (**E**) Cartoon view of an MCP protomer in the hexon, with domains colored and labeled. See fig. S8 (D and E) for details of hexon-hexon packing. (**F**) Cartoon view of an MCP penton, with one MCP protomer colored by domain. (**G**) Cartoon view of an MCP protomer in the hexon, with domains colored and labeled. Compared to the conformation in the hexon, in the penton the I domain undergoes an ∼30° rotation with respect to the other domains (fig. S8C). (**H**) View of the MCP penton with capsid latch protein (gp146; red) shown.

The MCPs of Goslar and PhiKZ show strong structural similarity, with an overall Cα root mean square deviation of 4.3 Å despite their relatively low sequence identity (25.8%) (fig. S8, A and B). Both Goslar and PhiKZ MCPs show a canonical HK97 fold (*43*, *44*), with an N-terminal arm (NTA), a peripheral domain (P domain), an axial domain (A domain), an insertion domain (I domain), and a short C-terminal arm (CTA; Fig. 2, D and E). MCP protomers in pentons and hexons have similar overall structure (fig. S8C), with each MCP protomer’s I domain packing against the I domain linkers and P domain of a neighboring protomer, and the A domains of all protomers packing on one another at the center of the assembly (Fig. 2, D and F). The extended NTA also forms a large interface with a neighboring MCP protomer, likely contributing to overall capsid stability. In the hexon, the short CTAs form a six-stranded β barrel at the very center of the assembly (Fig. 2D, inset); this structure is not observed in the penton. Overall, each MCP protomer in both the penton and hexon interacts with four other protomers in the assembly. The most obvious difference between the MCPs of the hexon and penton is a ∼30° twist/bend of each protomer’s I domain relative to the other domains (Fig. 2, E and G, and fig. S8C); this results in a pyramid-like architecture for the penton, compared to the flat architecture of the hexon. The distinctive structure of the penton is stabilized by the latch protein gp146 (Fig. 2H).

Side-by-side packing of MCP hexons (and pentons) is mediated by two sets of interactions. The major interaction involves the symmetric packing of two protomers’ I domains against one another, with residues Tyr 403, Ser 405, Arg 414, and Glu 421 forming a symmetric hydrogen-bond network (fig. S8, D and E). On either side of this interface, an extended loop comprising residues 211 to 229 reaches from each protomer to contact a nearby surface on the I domain of the opposite protomer (fig. S8E). These interactions also resemble how MCP hexons pack against one another in different phage capsids (*43*, *44*).

### The VBC

The recent cryo-EM structure of the *Pseudomonas*-infecting chimallivirus PhiKZ capsid revealed a VBC comprising seven proteins that form an interdigitated assembly at the inner surface of each non-portal vertex, docked on the inside of the central MCP penton (*41*). We similarly observe a fivefold symmetric multiprotein complex docked on the inner surface of the vertex (Fig. 3, A and C), comprising five proteins: gp42, gp59, gp65, gp66, and gp192 (Fig. 3D). Four of these proteins have clear homologs in the PhiKZ VBC (Fig. 3E), including gp42 (PhiKZ gp119), gp59 (PhiKZ gp91), gp65 (PhiKZ gp86), and gp66 (PhiKZ gp85). Goslar gp65 is much longer than its PhiKZ homolog (gp86) and includes a second folded domain not present in PhiKZ gp86 that occupies the same space as PhiKZ gp28 (Fig. 3, D and E). Goslar does encode a predicted homolog of PhiKZ gp28 (Goslar gp219), but we do not observe this protein in our cryo-EM map. We also do not observe homologs of two minor PhiKZ VBC proteins, gp162 and gp184.

**Fig. 3.**
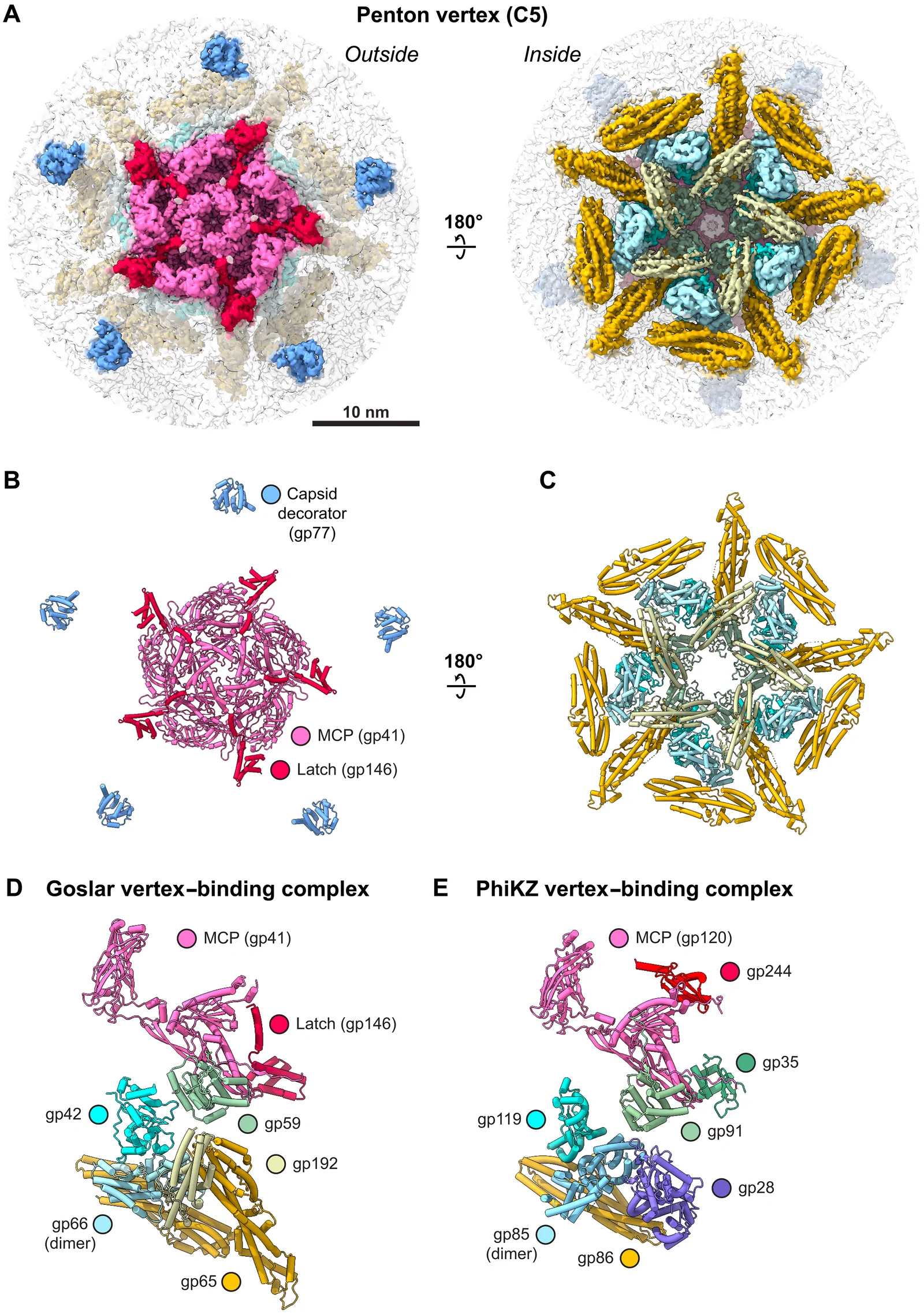
The VBC. (**A**) Two views of the 3.57-Å resolution C5 penton map. Left: View from outside the capsid, with density corresponding to the central MCP penton colored pink (surrounding MCP protomers transparent), the latch protein (gp146) red, and the capsid decorator protein (gp77) blue. Right: View from inside the capsid, with VBC colored as in (C). (**B**) Cartoon view of the MCP, latch, and decorator proteins as shown in (A). (**C**) Cartoon view of the VBC as shown in (A). (**D**) One unit of the VBC, with proteins colored as in (A) to (C) and labeled. (**E**) One unit of the VBC from the *Pseudomonas* phage PhiKZ (PDB 8Y6V) (*41*), with proteins homologous to Goslar VBC proteins colored identically to their homologs in (D). For clarity, minor components of the PhiKZ VBC without Goslar homologs (gp162 and gp184) are not shown. PhiKZ gp35 (dark green) decorates the outer surface of the PhiKZ capsid and does not have a Goslar homolog.

In addition to the above proteins, we observed additional density at low contour surrounding the VBC and packed against the capsid (fig. S9). This density resembles an extended α-helical bundle reminiscent of the PF12699 fold, of which there are several encoded by Goslar (see below). We were unable to assign this density.

### Architecture of empty capsids

In addition to intact Goslar virions, we also determined a 5.2-Å resolution icosahedrally averaged structure of empty Goslar capsids (fig. S4, A and B), which slightly outnumbered intact virions in our dataset [3033 intact virions versus 4535 empty capsids in dataset 1 (data S1)]. Compared to the intact virion capsid, the empty capsid has an overall identical symmetry and arrangement of MCP protomers (fig. S4, C to H). Empty capsids also have the penton-stabilizing latch protein (gp146) and the capsid decorator protein gp77. The strongest differences compared to intact virion capsids are that empty capsids lack both an identifiable portal and lack the internal VBC (fig. S4, D and G). These observations suggest that empty capsids represent spontaneous assemblies of the MCP, latch, and decorator that form without a portal, and are therefore defective particles unable to be filled with genomic DNA and attached to tails.

### Portal/neck architecture

Linking capsids to tails is the portal/neck complex, which comprises six different proteins. We determined a 3.8-Å structure of the C12-symmetric portal and upper neck (Fig. 4A), and a 4.2-Å resolution structure of the C6-symmetric lower neck (Fig. 4, B and C). The C12-symmetric region (Fig. 4D) comprises 12 copies each of the portal protein gp36, the adaptor protein gp233, and the N-terminal domain of the stopper protein gp50. The portal protein is positioned at the top of the assembly, with 12 copies arranged in a ring around a central channel that measures ∼74 Å at the top, decreasing to ∼60 Å at the bottom of the portal. The portal protein itself shares a similar overall architecture with other phage portal proteins, with the large wing domain, stem and clip domains extending downward from the wing domain, and C-terminal crown domain at the top of the assembly (Fig. 4D). The wing domain spans residues 5 to 519 and 643 to 777 of gp36, and the stem and clip domains span residues 520 to 642. These domains contact the adaptor protein gp233, which in turn packs against the N-terminal domain of the stopper protein gp50. The crown domain of gp36 spans residues 778 to 923 and reaches counter-clockwise (as viewed from above) across the top of the portal assembly to pack against the crown domains of three neighboring protomers. The portal protein gp36 and the adaptor protein gp233 show structural similarity to the T4 portal (gp20) and neck (gp13) proteins, respectively. Whereas the T4 gp13 dodecamer contacts the hexameric protein gp14, the Goslar adaptor protein gp233 contacts the dodecameric stopper protein gp50 (see below).

**Fig. 4.**
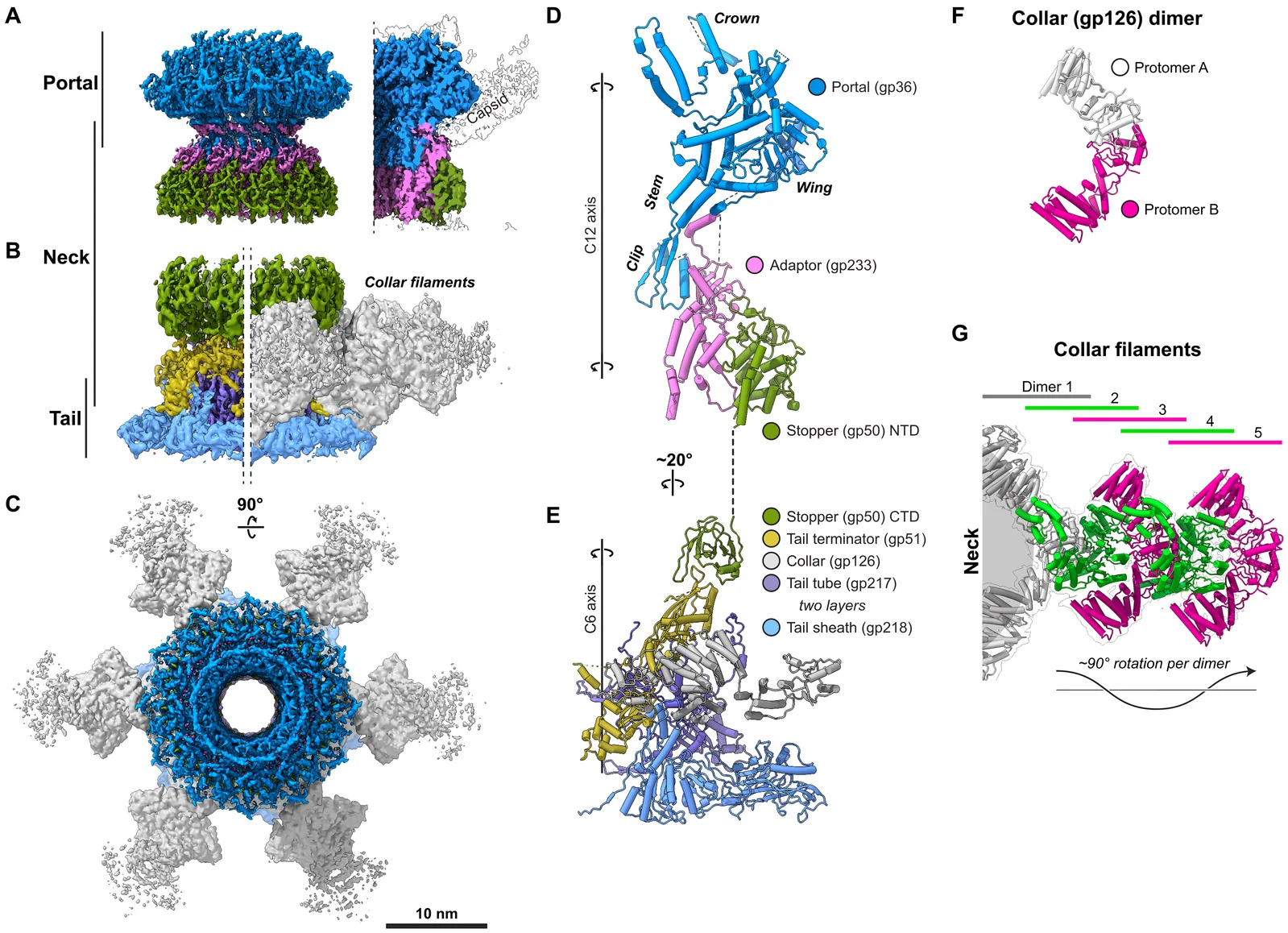
Portal and neck architecture. (**A**) Left: A 3.82-Å resolution map of the portal and upper neck (C12 symmetry) colored by protein; right: cutaway view of C12-symmetric map with density corresponding to the surrounding MCP capsomers shown in white. (**B**) Left: A 4.16-Å resolution map of the lower neck (C6 symmetry) colored by protein, with density for the collar subtracted; right: lower neck map showing density for the collar and collar filaments in gray. (**C**) Top-down view of combined portal/neck maps. (**D**) Cartoon view of the C12-symmetric portal and upper neck, with the portal protein (gp36) blue and domains labeled, adaptor protein (gp233) pink, and stopper protein (gp50) green. Only the N-terminal domain of the stopper protein is shown. (**E**) Cartoon view of the C6-symmetric lower neck, with stopper protein (gp50) green, tail terminator (gp51) yellow, collar protein (gp126) gray, tail tube protein (gp217; two layers shown) purple, and tail sheath protein (gp218) blue. Only the C-terminal domain of the stopper protein is shown. Only the inner-most dimer of the collar protein is shown. (**F**) Cartoon view of one dimer of the collar protein (gp126), with one protomer white and the second protomer magenta. (**G**) Top-down view of one collar filament, with the neck at left, and five dimers of the collar protein colored (from inside to outside) gray, green, magenta, green, and magenta.

Below the C12-symmetric upper neck is the C6-symmetric lower neck (Fig. 4E). The stopper protein (gp50) mediates this symmetry switch, showing C12 symmetry in its upper N-terminal domain that contacts the adaptor protein gp233 and a C6-symmetric alternating inside-outside conformation in its lower C-terminal domain. The C-terminal domain of the stopper protein binds the hexameric tail terminator protein gp51, which in turn binds the top layers of both the inner tail tube protein gp217 and the outer tail sheath protein gp218 (discussed below). Last, the tail terminator protein also interacts with the “collar” protein gp126. This protein forms a homodimer (Fig. 4F), and six gp126 homodimers form a ring around the tail terminator protein hexamer (Fig. 4E). Each gp126 homodimer in this collar structure serves as a docking point for a helical array of gp126 dimers that extends outward from the collar (Fig. 4G). We could visualize five gp126 homodimers in each of these “collar filaments,” with more visible at low contour levels in our map.

The portal/neck architecture of Goslar closely resembles that of PhiKZ, with the major exception that PhiKZ entirely lacks the collar filaments we observe around the Goslar neck. Instead, PhiKZ has a “whisker ring” comprising 6 copies of gp144 and 48 copies of gp34, for which there are not identified homologs in Goslar (*34*). The biological roles of the whisker ring in PhiKZ, and of the collar filaments in Goslar, are not known.

### Tail architecture

The Goslar tail is structurally similar to other contractile-tail phages and is composed of the tail tube protein (gp217) and the tail sheath protein (gp218) forming a C6-symmetric helical array (Fig. 5, A to D, and figs. S10 and S11). Gp218 is structurally similar to the tail sheath proteins of other phages (*45*, *46*), with an N-terminal central domain, an outer sheath domain, a C-terminal domain, and a C-terminal extension (Fig. 5E and fig. S10, A to F). To form the extended sheath structure, the outer sheath domain of one protomer interacts with the central domain of a neighboring protomer in the same layer, with the C-terminal domain of a protomer from a lower layer packing against the inside surface of these domains (fig. S10, G to I). The tail tube protein gp217 is also structurally related to other tail tube proteins (*47*, *48*), with a small N-terminal domain, conserved central domain, and C-terminal extension (Fig. 5F and fig. S11, A to F). The tail tube protomers form an extensive interlocking network, with the central domain of each protomer packing against the N-terminal domain of a neighboring protomer in the same layer, and each protomer’s C-terminal extension threading into the two layers above it (fig. S11, G to J). In our structure of the extended (uncontracted) Goslar tail, the tail tube and sheath adopt the same helical symmetry.

**Fig. 5.**
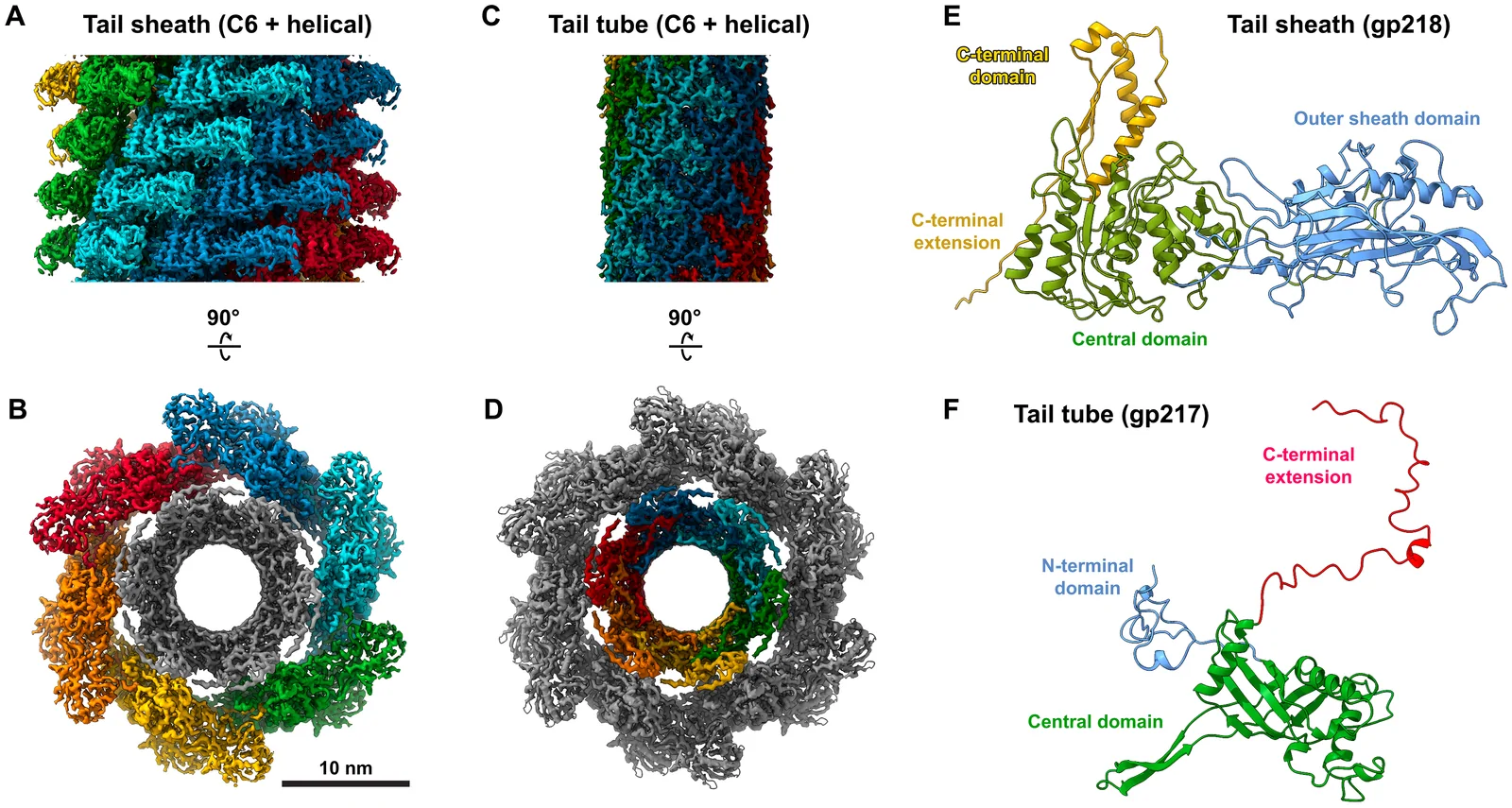
Tail architecture. (**A**) Side view of the tail sheath map, with each helical stack of tail sheath proteins (gp218) colored differently. (**B**) Top-down view of the combined tail tube/sheath map, with density corresponding to the tail sheath protein (gp218) colored. (**C**) Side view of the tail tube map, with each helical stack of tail tube proteins (gp217) colored differently. (**D**) Top-down view of the combined tail tube/sheath map, with density corresponding to the tail tube protein (gp217) colored. (**E**) Cartoon diagram of the tail sheath protein (gp218), colored by domain. See fig. S10 for details. (**F**) Cartoon diagram of the tail tube protein (gp217), colored by domain. See fig. S11 for details.

In the intact Goslar virion, there are 47 layers of the tail sheath protein, and 46 layers of the tail tube protein, similar to recent findings for PhiKZ (44 layers of tail sheath plus 43 layers of tail tube) (*34*). At the neck, the uppermost layer of the tail tube protein is capped by the tail terminator protein (gp51; Fig. 4E). Gp51 also packs against the upper-most layer of the tail sheath protein, which is adjacent to the second layer of the tail tube protein (Fig. 4E). At the baseplate (see next section), the lower-most layer of the tail tube protein is capped by the two-protein tail tube initiator complex: gp62, which is homologous to gp217, and gp203, which binds both gp62 and the hub protein gp48 (Fig. 6, A and B). The two-layer tube initiator complex is surrounded by the final two layers of the tail sheath protein, which pack against gp172 and gp63 in the inner baseplate (Fig. 6C).

**Fig. 6.**
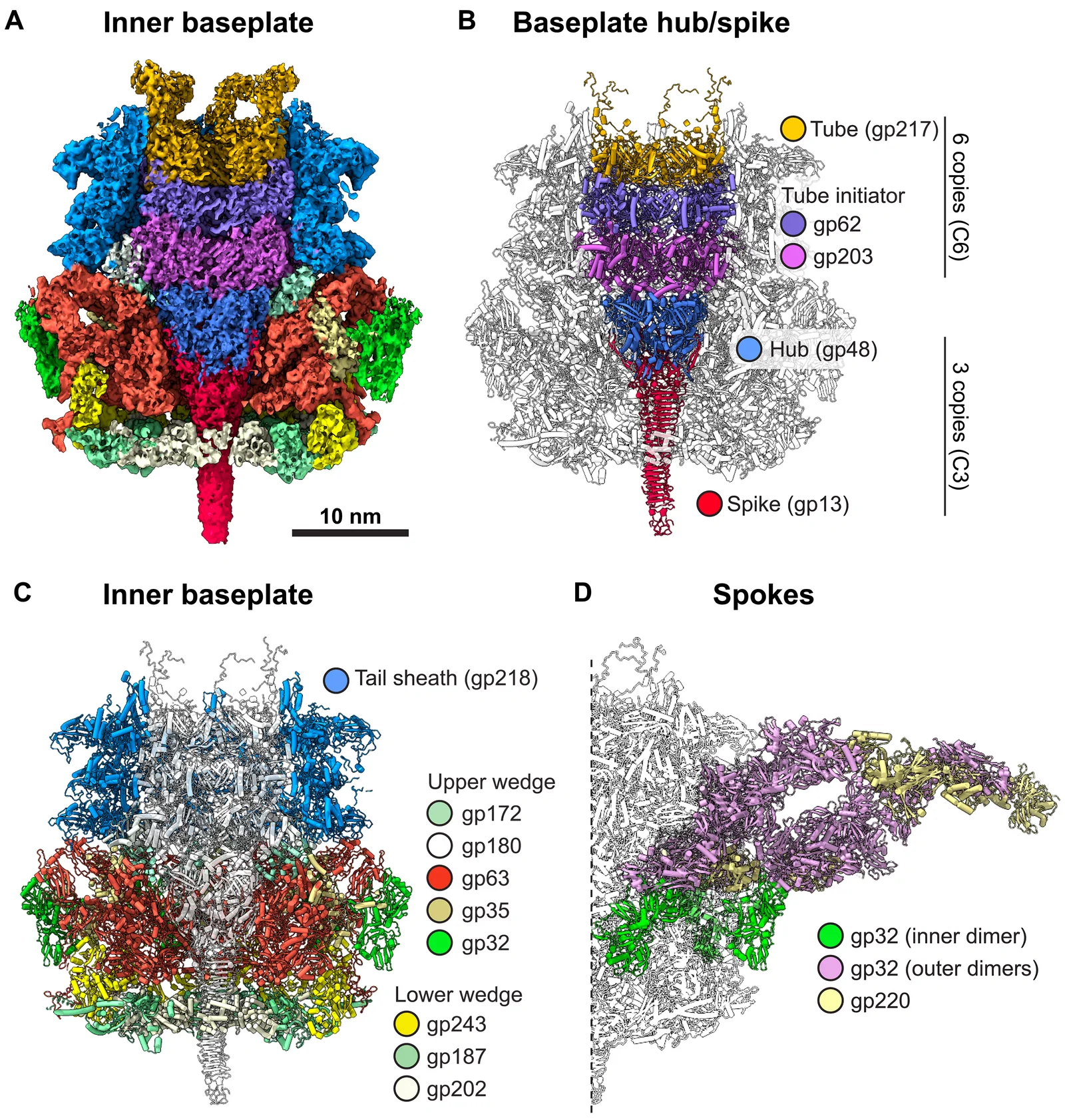
Baseplate architecture. (**A**) A 3.88-Å resolution cryo-EM map of the Goslar baseplate hub/spike and inner baseplate/wedges, colored by protein as indicated in (B) and (C). (**B**) Cartoon view with baseplate hub/spike proteins colored, and inner baseplate/wedge proteins shown in white. (**C**) Cartoon view with the inner baseplate/wedge proteins colored, and hub/spike proteins shown in white. See fig. S12 for details. (**D**) Cartoon view of the spokes connecting the inner and peripheral baseplates. Two spokes are shown, each with the inner-most dimer of gp32 [also shown in (B)] green, the outer four gp32 dimers pink, and two gp220 protomers light yellow. See fig. S13 (A to D) for details on spoke architecture and fig. S13 (E and F) for side and top views of the entire baseplate.

The related chimallivirus PhiKZ has been directly shown to have a contractile tail, with the contracted tail sheath measuring about half the length of the extended tail sheath (*32*, *39*). Similarly, we observe a few Goslar particles with contracted tails (fig. S10J). These particles were too few in number to perform structure determination on the contracted tails.

### Baseplate architecture

The Goslar baseplate is a large structure measuring nearly 100 nm across (Fig. 6 and figs. S12 and S13), and is made up of three main elements: (1) the baseplate hub/spike; (2) the inner baseplate/wedges; and (3) the peripheral baseplate. The inner and peripheral baseplates are connected by six “spokes,” creating an overall architecture that resembles a wheel.

From our 3.9-Å resolution C6-averaged map of the baseplate (Fig. 6A), we built atomic models for the baseplate hub/spike (Fig. 6B) and inner baseplate/wedges (Fig. 6C). The baseplate hub/spike comprises the spike protein gp13 (three copies, C3 symmetry), the hub protein gp48 (three copies, C3 symmetry), and the tail tube initiator complex (gp203 and gp62, six copies each, C6 symmetry). The bottom layer of the tail tube protein gp217 sits atop gp62 (Fig. 6B). Because the spike (gp13) and hub (gp48) proteins are C3 symmetric, we attempted to calculate a C3-averaged map of the baseplate to better visualize these proteins but could not resolve them. Therefore, these two proteins were not individually modeled, but, rather, an AlphaFold model of a 3:3 stoichiometry complex was manually placed and fit into the C6-averaged maps. Overall, the architecture of the baseplate hub/spike closely resembles that of phage T4, which also has a central spike (gp5 and gp5.4), a hub (gp27), and a two-layer tail initiator complex (gp48 and gp54) that links to the tail tube protein (gp19) (*49*).

The baseplate hub is surrounded by a sixfold symmetric inner baseplate structure (also termed the “wedges” by analogy with T4) comprising eight different proteins, which we assigned to “upper” and “lower” substructures (Fig. 6C). The upper wedge comprises five proteins (gp172, gp180, gp63, gp35, and gp32), and the lower wedge comprises three proteins (gp243, gp187, and gp202). Our cryo-EM map allowed us to assign all density of the baseplate hub/spike and inner baseplate/wedges and build and refine atomic models for all proteins.

The architecture of Goslar’s upper baseplate wedge is equivalent to the wedge structures in the baseplates of phage T4 and other contractile injection systems (fig. S12, A and B). In each of the six upper wedges, two copies of gp63 (equivalent to T4 gp6) and one copy of gp35 (equivalent to T4 gp7) form the structural core. Two proteins—gp172 (equivalent to T4 gp53) and gp180 (equivalent to T4 gp25)—extend upward to contact the first layer of the tail sheath protein gp218 (equivalent to T4 gp18). Last, gp35 extends outward to interact with a dimer of gp32, which forms the base of the “spoke” structures extending to the peripheral baseplate. This interaction is equivalent to T4 gp7 extending outward to contact gp8, which forms a structurally similar homodimer to Goslar gp32.

In addition to the upper baseplate wedge, Goslar has a lower baseplate wedge comprising four copies of gp243, two copies of gp187, and one copy of gp202 per wedge (fig. S12C). Gp243 forms a filament from two homodimers that associate end-to-end, which reaches downward from gp63 in the upper wedge; gp187 forms a homodimer that packs against gp243 and is braced by long hairpin extensions from both protomers of gp63 in the upper wedge; and gp202 packs against the inner surface of the gp187 dimer and contacts the spike protein gp13. The lower wedge is conserved in PhiKZ, where it is termed “hexagonal rings” 1 and 2 (*34*), but is not conserved in T4.

Extending outward from the inner baseplate/wedges are six spokes that connect to the peripheral baseplate, which is poorly resolved in our 3.9-Å resolution map. We performed a focused refinement using a mask covering one-sixth of the spoke and peripheral baseplate region, resulting in a 7.9-Å resolution map (fig. S6, D and E). By careful analysis of AlphaFold-predicted structures and predicted protein-protein interactions, we determine that the spoke comprises a filament of gp32 bound to gp220. Gp32 is predicted to assemble into a twofold symmetric homodimer that is structurally similar to T4 gp8, and we could build and refine one gp32 homodimer alongside gp35 in the inner baseplate/wedge (Fig. 6D and fig. S13, A and D). AlphaFold predicts that gp32 can also form a filament through end-to-end assembly of homodimers, and using the resulting predicted filament structure, we could place four additional gp32 homodimers (10 copies total) extending outward to connect the inner and peripheral baseplates. The overall structure of this filament and its role in linking the inner and peripheral baseplates resemble those of gp32 (and T4 gp8) homologs in the baseplates of several other phages, including gp105 from *Listeria* phage A511 (*50*) and the “arm segment” protein gp118 from Staphylococcus phage 812 (*51*). Last, we could also tentatively place two copies of gp220 against the gp32 filament, using an AlphaFold-predicted structure of a complex of these two proteins (fig. S13, A to F). Thus, the spokes comprise a repeating structure of gp32 and gp220. Our maps did not allow us to assign any other density in the peripheral baseplate.

As noted above, our structure of the Goslar baseplate shows strong similarity to the recent structure of PhiKZ, including the hub/spike and both upper and lower wedges (*34*). Also in agreement with our structure, each of the inner-peripheral baseplate spokes in PhiKZ comprise five dimers of the gp32 homolog gp139, plus two copies of the gp220 homolog gp27. In addition to these proteins, the higher resolution of the PhiKZ baseplate structure allowed modeling of several additional proteins, all of which have clear homologs in Goslar (table S1). Even so, major portions of the peripheral baseplate in both PhiKZ and Goslar remain unmodeled, leaving open questions about the detailed architecture of the peripheral baseplate and tail fibers, as well as any conformational changes associated with host recognition, tail sheath contraction, and genome injection.

### Mass spectrometry of Goslar virions identifies abundant virion-associated proteins

Chimalliviruses’ distinctive lifestyle, notably their mechanism of infection that involves injection of their genomic DNA into a lipid-enclosed vesicle, demands that these phages package and inject a suite of proteins alongside their DNA to mediate a successful infection. Chimalliviruses are known to inject the multisubunit vRNAP (*17*–*19*), high-copy proteins thought to make up the inner body (e.g., PhiKZ gp93 and its Goslar homolog gp58) (*12*, *13*, *52*), and other proteins with unknown functions (*13*). To identify the full set of proteins associated with the Goslar virion, we subjected our purified Goslar virions to liquid chromatography–tandem mass spectrometry (LC-MS/MS) with label-free quantitation (LFQ). Calibrating the MS2 peak area assigned to peptides from each protein with the known copy number of the MCP and tail tube/sheath proteins, we could roughly estimate (± about 50%) the per-virion copy number of all proteins detected in our dataset (see Materials and Methods) (Fig. 7, A and B, and data S3). By this measure, we detected 12 proteins present in >200 copies per virion that were not part of our atomic model of the Goslar virion (Fig. 7B). Two of these proteins were gp53 (∼330 copies per virion) and gp58 (∼2150 copies per virion), both of which contain a predicted PF12699 domain and are related to several PhiKZ proteins with the same domain (see below). The other 10 proteins we detect in >200 copies per virion (from highest to lowest abundance: gp23, gp158, gp214, gp88, gp22, gp208, gp61, gp209, gp7, and gp168) all lack a clearly predicted function from sequence or comparisons of their AlphaFold-predicted structures (fig. S14) to known proteins.

**Fig. 7.**
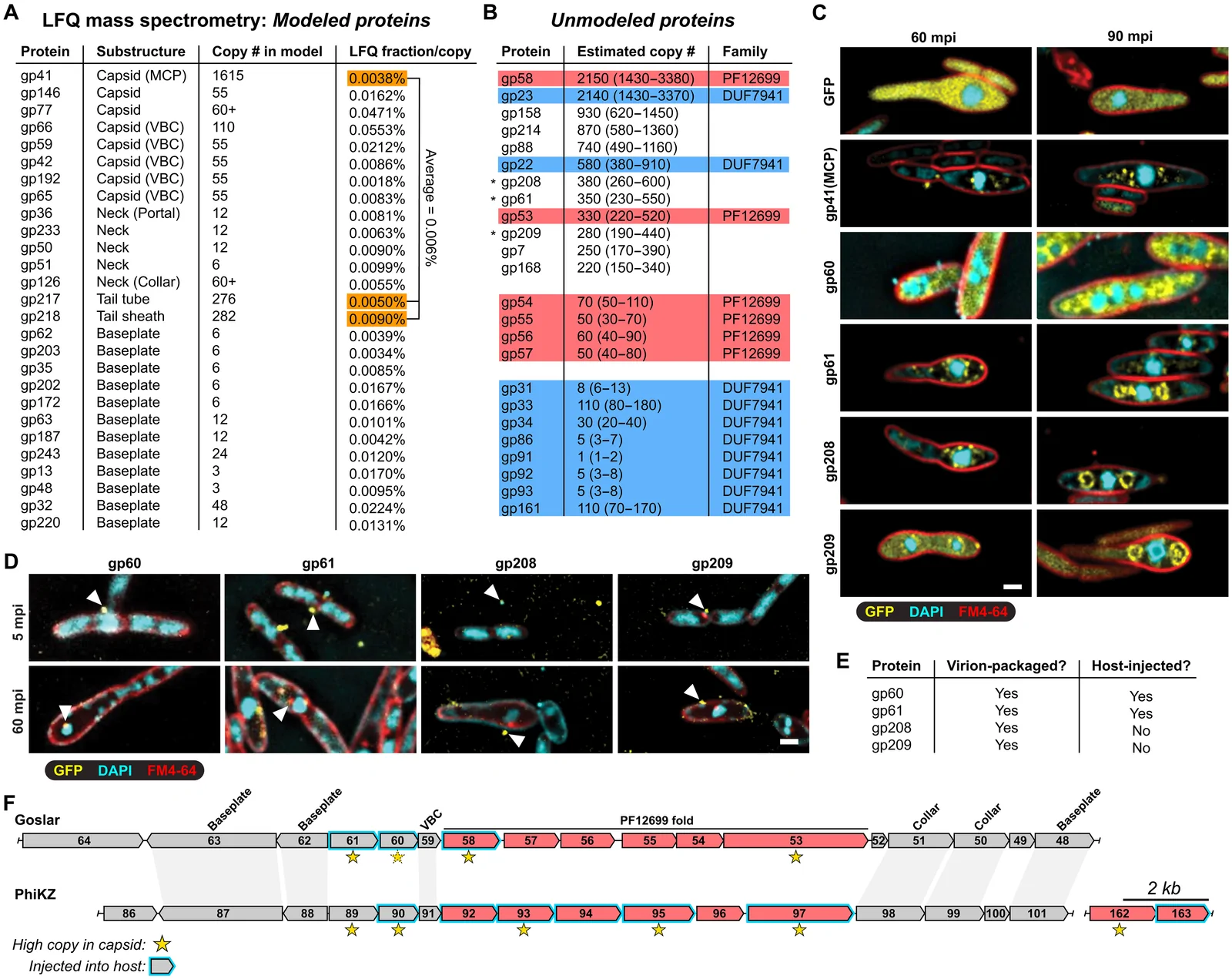
Mass spectrometry identifies abundant virion-associated proteins. (**A**) Results from LFQ mass spectrometry analysis of purified Goslar virions, showing 27 structural proteins (gp180 was not detected by mass spectrometry). See data S3 for details. (**B**) Select unmodeled proteins detected by mass spectrometry, and their estimated copy number per virion, calculated using an average LFQ fraction/copy of 0.006% (from gp41, gp217, and gp218 LFQ/copy) and the range from gp218 LFQ/copy (0.009%) to gp41 LFQ/copy (0.0038%). The 12 most-abundant proteins include two PF12699 proteins (gp58 and gp53) and two DUF7941 proteins (gp23 and gp22). Estimated copy number for all other PF12699 and DUF7941 proteins are also shown. See data S3 for details. (**C**) Localization of GFP-tagged Goslar proteins in infected cells, 60 and 90 mpi. From top to bottom: GFP, gp41-GFPmut1 (MCP), gp60-GFPmut1, GFPmut1-gp61, GFPmut1-gp208, and gp209-GFPmut1. GFP is yellow; 4′,6-diamidino-2-phenylindole (DAPI) (DNA) is cyan; FM4-64 (membranes) is red. Scale bar, 1 μm. See fig. S15 for details. (**D**) Fluorescence microscopy of Goslar virions with GFP-tagged proteins, 5 and 60 mpi. From left to right: gp60-GFPmut1, GFPmut1-gp61, GFPmut1-gp208, and gp209-GFPmut1. Arrowheads indicate the location of virions (on the cell surface) or EPI vesicles (within cells). Scale bar, 1 μm. See fig. S16 for details. (**E**) Summary of findings for virion incorporation and host injection for gp60, gp61, gp208, and gp209. (**F**) Schematic of the syntenic genomic regions of Goslar (NCBI NC_0481701.1) and PhiKZ (NCBI NC_004629.1) encoding a cluster of PF12699 proteins. Known homologs (*25*) are indicated in gray. Yellow stars indicate proteins present at high copy number in virions, as determined by mass spectrometry of Goslar virions (this study) and PhiKZ virions (*40*). Injected proteins are outlined in blue. See fig. S17 for predicted structures of all Goslar PF12699 proteins.

In all, we identified 87 Goslar proteins in our mass spectrometry dataset with a predicted copy number of five or more per virion. These proteins include all five subunits of the vRNAP that is packaged in the phage head (gp78, gp196, gp205, gp207, and gp210) plus several proteins annotated as tail or baseplate proteins that likely represent components of the peripheral baseplate/tail fibers not visible in our maps (e.g., gp25, gp29, and gp90; data S3). We also detected the nuclear shell protein chimallin (gp246) and proteins known to be chimallin-associated (e.g., gp176/ChmC), plus the tubulin-like protein PhuZ (gp234). These data show that our phage isolation method (including PEG precipitation and CsCl gradient centrifugation) also captured large protein assemblies including phage nucleus fragments and PhuZ filaments.

### Identification of virion-packaged and injected proteins

To determine whether the high-copy proteins we detect by mass spectrometry are packaged into virions and injected into subsequent host cells, we expressed green fluorescent protein (GFP) fusions of three high-copy proteins (gp61, gp208, and gp209) plus a fourth protein (gp60; present at an estimated ∼140 copies per virion) that is predicted by AlphaFold to bind gp61 (fig. S14I). We first examined these proteins’ localization in Goslar-infected cells. The localization of all four proteins resembled that of the MCP gp41, with puncta surrounding the phage nucleus at 60 min postinfection (mpi) and puncta clustering at the phage “bouquets” at 90 mpi (Fig. 7C and fig. S15). These data suggest that all four proteins are associated with virions but are not conclusive.

We next generated phage lysates from cells expressing each GFP fusion and infected wild-type MC1000 cells to test whether the proteins are detectably associated with infecting virions and whether they are injected into host cells. We found that all four proteins showed clear puncta representing Goslar virions adhered to host cells (Fig. 7D). We further found that gp60 and gp61, but not gp208 or gp209, were injected into host cells, colocalized with EPI vesicles in early infections (15 mpi), and formed a single focus on the phage nucleus in late infections (60 mpi) (Fig. 7D and fig. S16), consistent with prior observations of host-injected proteins in Goslar and PhiKZ (*12*, *13*, *42*, *52*). Overall, these data show that gp60, gp61, gp208, and gp209 are all virion associated and that gp60 and gp61 are injected into the host cell along with the phage genome (Fig. 7E).

In PhiKZ, a set of proteins with a common predicted fold characterized by the Pfam PF12699 domain are present at high copy number in the capsid and have been proposed to form a large internal assembly termed the inner body (*38*–*42*). These proteins (PhiKZ gp92, gp93, gp94, gp95, gp96, gp97, gp162, and gp163) are mostly encoded in a single operon (Fig. 7F), and most are also injected into the host cell upon infection (*13*, *42*, *52*). The Goslar proteins gp53 and gp58 also have a predicted PF12699 domain and are encoded in a region of the Goslar genome that is syntenic with the PhiKZ region that encodes gp92-gp97 (Fig. 7F). Like several PhiKZ PF12699 proteins, we also previously showed that Goslar gp58 is injected into the host cell (*12*). We performed AlphaFold structure predictions of the proteins encoded near gp53 and gp58 in the Goslar genome and found that gp54, gp55, gp56, and gp57 share the predicted PF12699 fold (fig. S17). We detect all of these proteins in our mass spectrometry: gp53 at ∼330 copies per virion, gp54 at ∼70 copies, gp55 at ∼50 copies, gp56 at ∼60 copies, gp57 at ∼50 copies, and gp58 at ∼2150 copies (Fig. 7B and data S3). Thus, while PhiKZ has several similar proteins at ∼100 to 200 copies per virion (*40*), Goslar has a single dominant PF12699 family member in gp58. The biological role of the injected PF12699 proteins, including whether their primary functions are within the capsid or in the EPI vesicle after injection into a host cell, is not known.

### Chimallivirus DUF7941 proteins form tail-associated filaments

We noticed that two of the highest-abundance proteins in the Goslar virion, gp22 (∼580 copies per virion) and gp23 (∼2140 copies), are paralogs of one another and share a common DUF7941 domain (DUF: domain of unknown function) (fig. S14, A and E). The annotated DUF7941 domain spans the entire length of gp22, which is predicted to comprise an N-terminal globular domain, a disordered linker, and a C-terminal globular domain (fig. S14E). Gp23 shares the N-terminal globular domain and disordered linker, but lacks gp22’s C-terminal globular domain. We could not identify any confident structural matches to gp22 or gp23 using the DALI (*53*) or FoldSeek (*54*) servers. Using HMM searches with the SeqHub web service (*55*) and AlphaFold structure predictions, we identified eight other Goslar proteins containing the DUF7941 module: gp31, gp33, gp34, gp86, gp91, gp92, gp93, and gp161 (Fig. 8A). In three of these (gp86, gp92, and gp161), an N-terminal DUF7941 module is followed by predicted carbohydrate binding, glycoside hydrolase, and/or leucine-rich repeat domains, suggesting a role for these proteins in host recognition (Fig. 8A and fig. S18). The DUF7941 module is found exclusively in chimalliviruses; for example, PhiKZ is predicted to have at least three DUF7941 proteins (gp126, gp127, and gp143), and the *Erwinia* phage RAY is predicted to have at least five (gp8, gp9, gp11, gp215, and gp218).

**Fig. 8.**
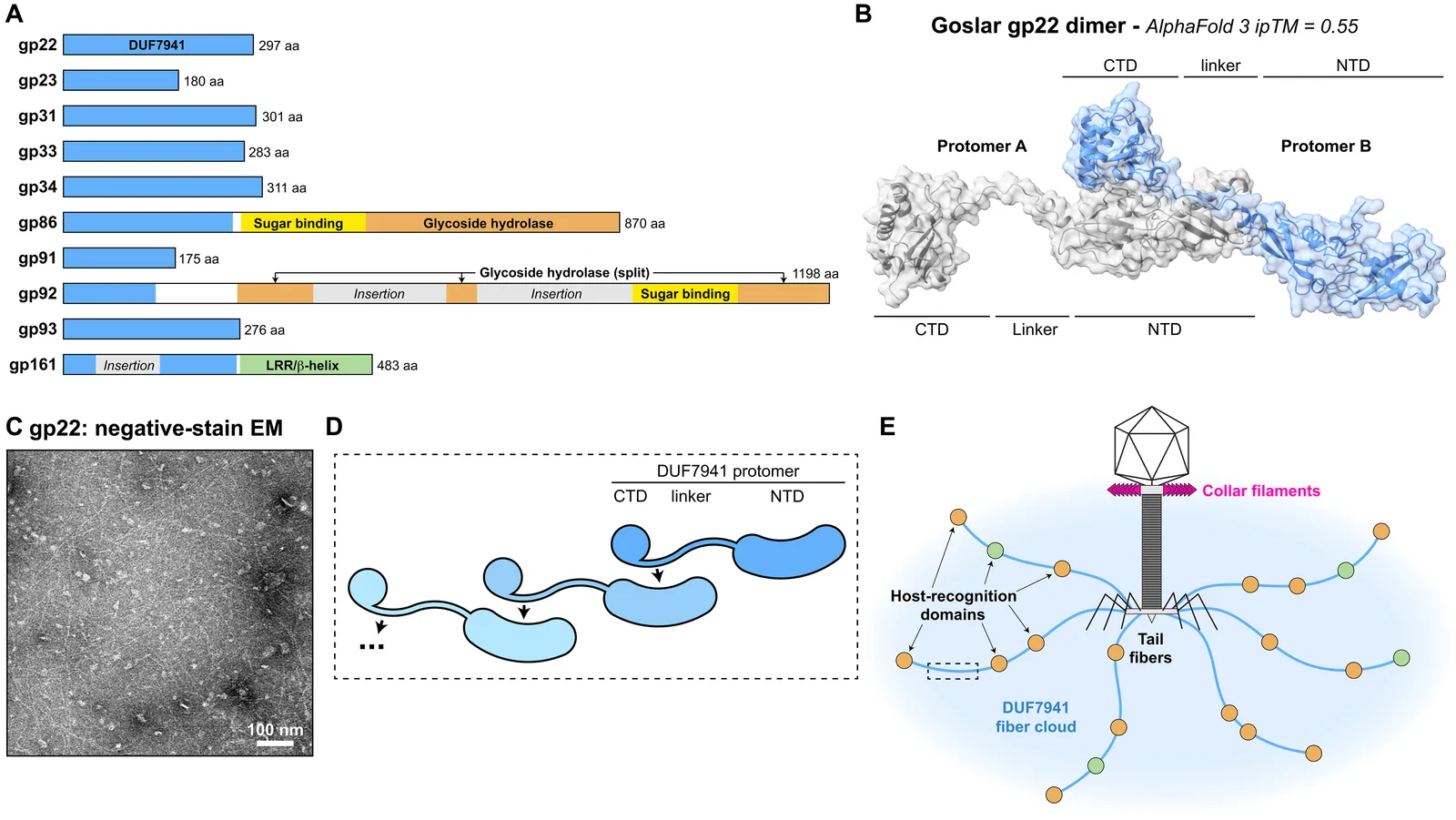
Identification of DUF7941 proteins in Goslar and model for oligomerization. (**A**) Domain schematics of 10 identified DUF7941 proteins in Goslar. DUF7941 domains are shown in blue, carbohydrate-binding domains in yellow, glycoside hydrolase domains in orange, leucine-rich repeat (LRR)/β-helix domains in green, and unknown domains in gray. AlphaFold predictions of all proteins are shown in fig. S18. (**B**) AlphaFold 3 predicted structure of a Goslar gp22 dimer. The disordered linker and CTD of one protomer (blue) is predicted to dock against the NTD of a second protomer (gray) in a head-to-tail orientation. See fig. S19 for further details and for predicted structures of a Goslar gp23 dimer. See fig. S20 for the predicted structure of a PhiKZ gp126-gp127 heterodimer and fitting of that model into cryo-EM density of the PhiKZ peripheral baseplate. (**C**) Negative-stain EM of purified Goslar gp22, showing extended filaments. See fig. S21 for additional images. (**D**) Model for head-to-tail assembly of DUF7941 domains. (**E**) Speculative model showing likely host-recognition elements in Goslar, with collar filaments shown in magenta, tail fibers in black, and the DUF7941 fiber cloud in blue, with associated carbohydrate binding/hydrolysis domains in orange and LRR/β-helix domains in green.

Curiously, AlphaFold predictions suggest that Goslar gp22 could form head-to-tail oligomers through binding of one protomer’s disordered region to the N-terminal globular domain of another protomer, forming a contiguous intermolecular β sheet (Fig. 8B and fig. S19, A and B). While Goslar gp23 lacks the C-terminal globular domain of gp22, it is also predicted to form head-to-tail oligomers (fig. S19, C and E). We used AlphaFold to predict homo- or hetero-oligomer formation between each pair of DUF7941 proteins in Goslar and identified a network of potential interactions, suggesting that these proteins may form extended, heterogeneous filaments (fig. S19F). Relatedly, we found that the PhiKZ DUF7941 proteins gp126 and gp127 are confidently predicted by AlphaFold to form a head-to-tail dimer (fig. S20, A and C). In a recent structure of the intact PhiKZ virion, the DUF4391 protein gp127 is partially modeled adjacent to the outermost dimer of gp139 (homologous to Goslar gp32) (*34*). In the published PhiKZ structure, only the gp127 NTD is modeled (fig. S20D), but we could model a full gp126-gp127 heterodimer into unexplained density in the PhiKZ outer peripheral baseplate map (fig. S20, E and F). Therefore, while no density for DUF4391 proteins is observable in our less well-defined maps of the Goslar peripheral baseplate (fig. S13), we propose that chimallivirus DUF7941 proteins universally associate with the peripheral baseplate.

To test whether DUF7941 proteins can form oligomers, we expressed and purified Goslar gp22. Because *E. coli* lysates containing recombinantly expressed His_6_-gp22 were extremely viscous, we performed a single-step purification using Ni^2+^ affinity chromatography in buffers containing 2 M urea (fig. S21A). After exchange into a buffer without urea, we applied the purified protein to an EM grid and examined it by negative-stain EM. We observed filaments and filament bundles up to several hundred nanometers in length (Fig. 8C and fig. S21B). Combined with our AlphaFold predictions suggesting that DUF7941 proteins form head-to-tail oligomers, we propose that chimallivirus DUF7941 proteins form a “cloud” of extended, flexible filaments that facilitate initial host recognition (Fig. 8, D and E). On the basis of the overall copy number estimated from mass spectrometry (more than ∼2500 DUF7941 domain proteins per Goslar virion), protomer spacing predicted by AlphaFold (∼7 nm per protomer), and the likely number of peripheral baseplate anchoring sites (*6*), we calculate an average DUF7941 filament length of nearly 3 micrometers in Goslar. Thus, the DUF7941 filament cloud would be expected to dramatically increase the ability of each virion to encounter and bind host cells.

## DISCUSSION

Here, we use cryo-EM to define the overall molecular architecture of the chimallivirus Goslar, with our final model comprising 2888 polypeptide chains from 28 different structural proteins. Our maps of individual phage substructures ranged from ∼3.2 to ∼4.2 Å in resolution, allowing us to build and refine atomic models for most structural proteins. The assistance of modern artificial intelligence tools including AlphaFold (*37*) and ModelAngelo (*36*) was crucial for initial assignment of low-resolution density, initial model building, and predictions of protein-protein interactions and complex architectures within each substructure. Overall, the architecture of Goslar is consistent with that of the related phage PhiKZ (*34*) and with other tailed phages, with a *T* = 27 icosahedral capsid, 12-fold symmetric portal, helical tail assembly, and baseplate with central spike (threefold symmetric), inner baseplate/wedges (sixfold symmetric), and an extended peripheral baseplate (sixfold symmetric). While we could assign density for the spokes connecting the inner and peripheral baseplates, our maps were unable to resolve the full peripheral baseplate and tail fibers.

Our cryo-EM model and quantitative mass spectrometry analysis of purified Goslar virions reveal several unique properties of this large-genome phage. First, we define an assembly of collar filaments extending from the portal-neck region and made up of an extended array of gp126 dimers. The role of these filaments is not known: They could act as an environmental sensor akin to the phage T4 whiskers (*56*, *57*) or interact with host flagella similarly to the capsid fibers of phage 7-1-1 (*58*). There is strong evidence supporting the idea that PhiKZ and related chimalliviruses use the host flagellum for initial recognition, including the observation that these phages tend to infect near the cell pole (*59*) and that phage-resistant mutants often affect the flagellum (*60*, *61*). Notably, the collar filaments we observe associated with the Goslar neck region are completely distinct from the whisker ring recently reported as surrounding the neck region of the related phage PhiKZ (*34*).

Another distinctive feature of Goslar—which is shared with PhiKZ—is the presence of high-copy virion associated proteins that have the uncharacterized PF12699 domain. In PhiKZ, these proteins have been proposed to make up the inner body, a large structure that has been observed in the PhiKZ head by low-resolution EM studies (*40*–*42*), but notably not observed in recent high-resolution studies of the same phage (*34*). We do not observe the inner body in our cryo-EM micrographs or 3D reconstructions of Goslar, but purified Goslar phages do contain thousands of copies of PF12699 domain proteins per virion. Gp58 is most abundant, with an estimated 2000+ copies per virion, and we also detect dozens to hundreds of copies of each of the structurally related proteins gp53, gp54, gp55, gp56, and gp57. Many PF12699 proteins (including Goslar gp58) are known to be injected into the EPI vesicle formed upon initial infection of a host (*12*, *13*, *42*, *52*). The biological roles of these proteins remain a key unsolved mystery of the chimallivirus life cycle.

Our mass spectrometry also identified a family of chimallivirus-specific proteins characterized by the DUF7941 domain. We find that Goslar virions incorporate hundreds of copies of the DUF7941 proteins gp22 and gp23, and we also detect eight additional DUF7941 proteins associated with Goslar virions. Our AlphaFold predictions and negative-stain EM of gp22 indicate that the DUF7941 module can form head-to-tail oligomers. Combined with our finding that several Goslar DUF7941 proteins encode additional domains associated with host recognition, we propose that these proteins associate with the peripheral baseplate and form a “DUF7941 fiber cloud” extending up to several micrometers from the phage itself.

Our work sets the stage to answer a range of pressing questions regarding chimallivirus infection and development. Aside from determining the relative contributions of different elements—collar fibers, tail fibers, and newly identified DUF7941 fibers—to host recognition, a key question is how these phages inject their genomes into a lipid vesicle in their host. The tail architecture of Goslar is similar to other tailed phages, and we observe occasional virions with contracted tail sheaths in our cryo-EM micrographs (fig. S9H). Key next steps will be to determine in detail the structural changes that occur upon tail sheath contraction: how host cells are penetrated and how the lipid bilayer comprising the EPI vesicle is shaped upon injection of the genome and associated proteins.

## MATERIALS AND METHODS

### Phage Goslar preparation

Bacteriophage Goslar was originally acquired from J. Wittman at the DSMZ (*28*). For phage propagation, 100 μl of medium-titer viral lysate (10^8^ to 10^9^ PFU/ml) was incubated with 0.5 ml of *E. coli* MC1000 cells for 10 min, plated on LB agar plates after mixing with 4.5 ml of 0.35% LB top agar, and incubated overnight at 37°C. Lysis of host cells were observed by plate clearing as compared to a control plate containing only MC1000 cells. Viral particles were harvested by the addition of 5 ml of LB media, incubation overnight at 4°C, and followed by gentle scraping using a sterile cell spreader. After collection, the lysates were subjected to a low-speed spin (3220*g* for 10 min) followed by filtration through a 0.45-μm syringe filter. The resulting supernatant was treated with deoxyribonuclease I (DNase I, 1 μg/ml) and ribonuclease A (1 μg/ml) for 1 hour at 37°C, followed by addition of 2 ml (0.4 ml per 1 ml of phage lysate) phage precipitating solution (30% PEG molecular weight 8000 and 3.3 M NaCl) and kept at 4°C overnight. This was followed by centrifugation at 10,000*g* for 20 min to sediment phage particles. The pellet was resuspended in phage buffer [10 mM tris-HCl (pH 7.5), 10 mM MgSO_4_, 68 mM NaCl, and 1 mM CaCl_2_], mixed with solid CsCl (0.55 g per 1 ml of resuspended phages), and spun in an ultracentrifuge (55,000 RPM, 12 hours, using a Beckman-Coulter TLS-55 Rotor). The opalescent phage “band” was extracted with a syringe, buffer-exchanged to phage buffer, and concentrated by ultrafiltration (Amicon Ultra, EMD Millipore) to a final concentration of 10^10^ PFU/ml for mass spectrometry.

### Cryo-EM sample preparation and data collection

Because of low particle density on grids when using CsCl-purified phages, we skipped PEG precipitation and ultracentrifugation for cryo-EM samples. Instead, 3 μl of filtered phage lysate was directly applied to Carbon-coated Lacey-Carbon grids (Electron Microscopy Sciences) and imaged using a Titan Krios microscope (Thermo Fisher Scientific, operated at 300 kV) equipped with a K3 direct electron detector (Gatan) and a Biocontinuum Energy Filter (Gatan). Three cryo-EM datasets were collected. Dataset 1 consisted of 4224 movies collected in super-resolution mode under a nominal magnification of 33,000× (corresponding to a pixel size of 1.3 Å) with a total dose of 35 e^−^/Å^2^ and a defocus range of −0.5 to −4 μm. Datasets 2 (5677 movies) and 3 (7841 movies) were collected in counting mode under a nominal magnification of 64,000× (corresponding to a pixel size of 1.4 Å) with a total dose of 50 e^−^/Å^2^ with defocus range of −1 to −3 μm. All data-collection statistics are reported in data S1 (dataset 1) and data S2 (datasets 2 and 3).

### Cryo-EM data processing

#### Intact Goslar virions

All cryo-EM processing was performed with cryoSPARC version 4.7 (*35*). Intact virions were manually picked from dataset 1, with picking performed such that particles were centered on capsid’s portal vertex. Particles were aligned using 2D classification and then recentered to the midpoint of the virion along the tail for extraction. Particles were extracted with a 3600-pixel box and then Fourier-cropped to 800 pixels. For 3D reconstruction, 3033 particles were subjected to homogenous reconstruction followed by local CTF correction and then local refinement, yielding a 19.46-Å resolution map of the intact Goslar virion (fig. S1).

#### Empty capsid

From dataset 1, ∼100 empty capsids were manually picked and subjected to 2D classification to generate templates for automated particle picking. Template-based picking followed by multiple rounds of 2D classification yielded a final set of 4535 particles, which were extracted with an 1800 pixel box size and Fourier-cropped to 960 pixels (the maximum particle size possible given the memory limits of the NVIDIA RTX A5000 GPUs used for refinement). After Ab Initio reconstruction, multiple rounds of homogeneous refinement and global CTF correction were performed with icosahedral (I1) symmetry to account for the large particle size. For homogeneous refinement, the following nondefault parameters were used: fit spherical aberration, fit tetrafoil, fit anisotropic mag., and do EWS correction (fig. S4).

To identify the portal vertex, the particles were symmetry expanded, rotated to align particles on the C5 axis, and reextracted with a 600-pixel box, and then duplicate particles were removed and a homogeneous reconstruction was performed with a final set of 54,368 symmetry-expanded particles. We then performed 3C classification with 2 to 10 3D classes. No portal vertices were identified in empty capsids by this analysis (fig. S4B).

#### Capsid

From datasets 2 and 3, capsids attached to tails were initially picked using the “blob picker” job in CryoSPARC with particle diameter 1000 to 1800 Å, followed by iterative 2D classifications to define a 5214-particle set. Particles were extracted with a 1700-pixel box size, Fourier-cropped to 800 pixels, and then subjected to global CTF refinement followed by homogenous refinement with icosahedral (I1) symmetry imposed. This yielded a 6.28-Å resolution icosahedral map for the phage capsid (figs. S2 and S3, A and B). Particles were symmetry expanded (I1) and recentered on either the C3 (hexon) or C5 (penton) symmetry axes using the volume alignment job in cryoSPARC. For the reconstruction of the C3 hexon map, C3-centered particles were reextracted with a 480-pixel box size, and duplicate particles were removed. The remaining particles were subjected to local refinement with C3 symmetry, yielding a 3.57-Å resolution map, which was iteratively improved by several rounds of local CTF refinement and local refinement (figs. S2 and S3, C and D).

C5-centered particles were used to reconstruct an initial volume with the “homogenous reconstruct only” job in cryoSPARC. The resulting volume was used for a 3D classification job with 14 3D classes to isolate the unique portal vertex class (see below). Particles assigned to nonportal vertex classes were combined and subjected to iterative local refinement and local CTF refinement with C5 symmetry (figs. S2 and S3, E and F).

#### Portal and neck

Symmetry-expanded capsid particles assigned to the portal vertex class in 3D classification (see above) were reextracted with a box size of 640 pixels, symmetry expanded (C12), and then subjected to local refinement—This represents the upper region of the portal-neck assembly (figs. S2 and S3, G and H). The same particles were symmetry expanded (C6) and subjected to local refinement—This represents the lower neck region (figs. S2 and S3, I and J).

#### Tail

From datasets 2 and 3, tail particles were picked using the filament tracer job in cryoSPARC with filament diameter set to 300 Å and particle separation distance set to 150 Å. Initial particle picks were curated by 2D classification, resulting in a final set of 47,640 particles. These particles were extracted with a box size of 420 pixels, followed by helical refinement with helical twist 21.48°, helical rise 39.16 Å, and symmetry order 4 to yield a 3.39-Å resolution tail map (fig. S5, A to C). Particles were symmetry expanded, and then local refinement was performed with focused masks around either the tail tube or the tail sheath, yielding a tail tube map at 3.22-Å resolution (fig. S5, D to F) and a tail sheath map at 3.36-Å resolution (fig. S5, G to I).

#### Baseplate

From datasets 2 and 3, ∼100 baseplate particles were manually picked and subjected to 2D classification to generate templates for automated particle picking. Automatically picked particles were curated by 2D classification, yielding a final set of 5539 baseplate particles. Particles were extracted with a box size of 800 pixels and Fourier-cropped to a box size of 600 pixels, and then homogeneous reconstruction was performed with C6 symmetry to obtain an initial 4.35-Å resolution baseplate map. Density representing the lower tail was subtracted, and then the particles were symmetry expanded (C6) and subjected to iterative local refinement and local CTF refinement to yield the final 3.88-Å resolution baseplate map (fig. S6, A to C). To visualize the spokes and peripheral baseplate, a mask encompassing one-sixth of this region was generated and used as a focus mask with symmetry-expanded particles. This resulted in a 7.88-Å resolution peripheral baseplate map (fig. S6, D and E).

### Model building and refinement

Initial atomic models for several proteins were built by ModelAngelo (*36*). For well-annotated structural proteins such as the portal, sequence-based ModelAngelo was run to generate an initial model, which was validated by Alphafold3 predictions (*37*), followed by manual building in COOT (*62*) and refinement using PHENIX (*63*). For substructures where candidate proteins were unknown, sequence-free ModelAngelo was performed, and then the Modelangelo-assigned sequences were compared against the Goslar proteome using BLAST to identify candidate proteins. Candidates were validated by docking AlphaFold3-generated models (*37*) and by leveraging predicted protein-protein interactions with neighboring proteins, generated by AlphaFold3 copredictions. All models were manually rebuilt in COOT (*62*) and refined in phenix.refine (*63*) with strict noncrystallographic symmetry constraints for symmetry-related protomers, secondary structure restraints, and reference-model restraints (using confident AlphaFold3 models as reference models when available). For the C3-symmetric hub/spike complex, an AlphaFold model of a 3:3 complex of gp48 (hub) and gp13 (spike) was manually docked into the C6-symmetric baseplate map (orientation informed by an AlphaFold prediction of a 6:3 complex of gp203 and gp48) and rigid-body refined. For the baseplate spoke/peripheral baseplate region, manually docked AlphaFold models for gp32 (dimers) and gp220 (separate N- and C-terminal domains) were rigid-body refined using the 7.88-Å resolution spoke/peripheral baseplate map. All refinement statistics are reported in data S1 and S2.

A final composite model of the full Goslar virion (Fig. 1A) was generated by progressively building overlapping large substructures (full capsid with portal, portal with neck, neck with full tail, and tail with baseplate) and manually docking these substructures to one another and then to the asymmetric Goslar virion map. Structures were visualized in UCSF ChimeraX (*64*).

### Bioinformatics and structure prediction

To define Goslar proteins with PF12699 or DUF7941 domains, we used annotations available at InterPro (https://ebi.ac.uk/interpro/) (*65*) and examined sequences using SeqHub (https://seqhub.org; Tatta Bio) (*55*). For structure prediction of unmodeled proteins including PF12699 and DUF7941 proteins, predictions were performed using AlphaFold3 (*37*) through the AlphaFold server (https://alphafoldserver.com). Structures were visualized in UCSF ChimeraX (*64*), and PAE plots were generated using PAE Viewer (https://pae-viewer.uni-goettingen.de) (*66*).

### Mass spectrometry sample preparation

For mass spectrometry, CsCl-purified Goslar phage samples were desiccated and reconstituted in 200 μl of 6 M guanidine-HCl. The samples were then boiled for 10 min followed by 5-min cooling at room temperature. The boiling and cooling cycle was repeated a total of three cycles. Proteins were precipitated with addition of 1.8 ml of methanol final concentration, 90% methanol (v/v) followed by vortexing and centrifugation at 14,000 RPM on a benchtop microcentrifuge for 10 min. The soluble fraction was removed by flipping the tube onto an absorbent surface and tapping to remove any liquid. The pellet was suspended in 200 μl of 8 M urea in 100 mM tris (pH 8.0). TCEP [tris(2-carboxyethyl)phosphine] was added to a final concentration of 10 mM, 2-chloroacetamide was added to a final concentration of 40 mM, and the solution was vortexed for 5 min. Three volumes of 50 mM tris (pH 8.0) were added to the sample to reduce the final urea concentration to 2 M. Mass spectrometry–grade trypsin was added in a 1:50 ratio of trypsin to protein, and the solution was incubated at 37°C for 12 hours. The solution was then acidified using trifluoroacetic acid (0.5% final concentration) and mixed. The sample was desalted using C18-StageTips (Thermo Fisher Scientific). The final peptide concentration of the sample was measured using a BCA protein assay.

### Mass spectrometry data-dependent acquisition

One microgram of each sample was analyzed by ultrahigh pressure LC (UPLC) coupled with MS/MS (LC-MS/MS) using nanospray ionization. The nanospray ionization experiments were performed using a TimsTOF 2 HT hybrid mass spectrometer (Bruker) interfaced with nanoscale reversed-phase UPLC (Evosep One). The Evosep method 30 SPD (30 samples per day) was used with a 15 cm–by–150 μm reverse-phase column packed with 1.5-μm C18-beads (PepSep, Bruker) at 58°C. The analytical columns were connected with a fused silica inside diameter (ID) emitter (10-μm ID; Bruker Daltonics) inside a nanoelectrospray ion source (Captive spray source; Bruker). The mobile phases comprised 0.1% formic acid (FA) as solution A and 0.1% FA/99.9% acetonitrile as solution B. The mass spectrometry settings for the TimsTOF 2 HT were as follows: The Parallel Accumulation–Serial Fragmentation (PASEF) method was used for standard proteomics. The values for mobility-dependent collision energy ramping were set to 95 eV at an inverse reduced mobility (1/k_0_) of 1.6 V s/cm^2^ and 23 eV at 0.73 V s/cm^2^. Collision energies were linearly interpolated between these two 1/k_0_ values and kept constant above or below. No merging of thermal ionization mass spectrometry scans was performed. Target intensity per individual PASEF precursor was set to 20,000. The scan range was set between 0.6 and 1.6 V s/cm^2^ with a ramp time of 166 ms. Fourteen (14) PASEF MS/MS scans were triggered per cycle (2.57 s) with a maximum of seven precursors per mobilogram. Precursor ions in a mass/charge ratio range between 100 and 1700 with charge states ≥3+ and ≤8+ were selected for fragmentation. Active exclusion was enabled for 0.4 min (mass width 0.015 Th, 1/k_0_ width 0.015 V s/cm^2^).

### Mass spectrometry data analysis and normalization

Peptide identification and quantification was carried out using Peaks Studio 12.5 (Bioinformatics Solutions Inc.) (*67*), using sequence databases for the *E. coli* and phage Goslar proteomes; only peptides assigned to Goslar proteins were further processed. LFQ peak areas for each peptide were summed to generate total LFQ peak area per protein, and then this value was divided by the predicted molecular weight of each protein to generate LFQ area/mass for each protein. The total LFQ area/mass was calculated by summing across all detected Goslar proteins, and then each protein’s LFQ area/mass was divided by this sum to generate LFQ copy number fraction for each protein. For structural proteins with a known copy number per virion, the LFQ copy number fraction was divided by this number to generate the LFQ fraction per copy. To estimate the copy number per virion of proteins not detected in our structures, we used the LFQ fraction per copy for three known high-copy proteins: MCP (gp41) at 0.00382% per copy, tail tube (gp217) at 0.00502% per copy, and tail sheath (gp218) at 0.00901% per copy to define an estimated median LFQ fraction per copy of ∼0.006% ± ∼0.003%. Other structural proteins like those in the portal vertex complex were not used for this calculation because of uncertainty about the total copy number of these proteins per virion. The estimated copy number using the mean LFQ fraction per copy of 0.006% is reported in Fig. 7A; upper and lower estimates for each protein are available in data S3).

### Infections and microscopy

#### Plasmid construction and bacterial transformation

Selected Goslar genes were closed as fusions to GFPmut1 at either the N or C terminus and expressed from vector pDSW206 (*28*) modified to include a second RBS, “TTTAAGAAGGAGAAATTCACC,” to drive increased protein expression compared to the standard vector. One microliter of plasmid (20 μg/ml) was added to 30 to 50 μl of electroporation-competent *E. coli* MC1000 cells (strain EA258; prepared by washing in 10% glycerol) and electroporated with 2.5 kV. After transformants recovered in 1 ml of SOC at 37°C for 60 min, they were spread on LB plates with ampicillin (100 μg/ml) and incubated overnight at 37°C.

#### Fluorescence microscopy

Fluorescence microscopy experiments were performed in biological triplicate. Host cells were suspended in LB at optical density at 600 nm (OD_600_) of 0.6 as measured by nanodrop. Five microliters was spread on the surface of 1% agarose, 25% LB imaging pads [containing ampicillin (100 μg/ml) plus 10 or 100 μM isopropyl β-d-1-thiogalactopyranoside (IPTG) for induction of GFP fusion protein expression] in single-well concavity glass slides. After the slides were incubated for 1.5 to 2 hours at 37°C without coverslips in a humidor, 10 μl of Goslar lysate (∼10^10^ PFU/ml) was spread onto the imaging pads and the pads were incubated further at 37°C until the desired infection time point. For fluorescence microscopy, pads were stained with 8 μl of dye mix containing 4′,6-diamidino-2-phenylindole (25 μg/ml) and FM4-64 (3.75 μg/ml) at room temperature ∼5 min before imaging. Cells were imaged with 5 to 12 slices in the *z* axis at 0.2- or 0.3-μm increments. All microscopy was performed on a DeltaVision Elite Deconvolution microscope (Applied Precision, Issaquah, WA, USA) via DeltaVision SoftWoRx (version 6.5.2). Images were deconvolved in DeltaVision SoftWoRx (version 6.5.2).

### DNA cloning and protein expression

For protein expression, Goslar gp22 (NCBI:YP_009820707.1) was cloned into UC Berkeley Macrolab vector 2-BT (Addgene #29666) to generate an N-terminal TEV protease-cleavable His_6_-tagged construct. Proteins were expressed in *E. coli* Rosetta2 pLysS (EMD Millipore) by growing cells at 37°C to an OD_600_ of 0.6 to 0.8, followed by induction with 0.33 mM IPTG. Cultures were incubated overnight at 20°C with shaking. After 14 to 16 hours, cells were collected by centrifugation, and pellets were resuspended in ice-cold resuspension buffer containing 20 mM tris-HCl (pH 7.5), 300 mM NaCl, 10 mM imidazole, 10% glycerol, and 2 mM β-mercaptoethanol, plus protease inhibitors [aprotinin (2 μg/ml), leupeptin (0.5 μg/ml), pepstatin (0.7 μg/ml), and 1 mM phenylmethylsulfonyl fluoride] and DNase I (5 μg/ml). Resuspended cells were lysed by sonication (Branson Sonifier), and the lysate was clarified by centrifugation. The protein was purified using Ni^2+^ affinity chromatography (Ni–nitrilotriacetic acid Superflow, Qiagen), with one wash in resuspension buffer plus 2 M urea to disrupt large protein aggregates. Protein was eluted with resuspension buffer plus 250 mM imidazole.

### Negative-stain EM

Five microliters of purified gp22 protein was applied to a glow discharged 400 mesh Copper formvar/Carbon EM grid (Ted Pella, #01754-F) for 5 min. The grid was washed three times with 0.22 μm of filtered double-distilled water and incubated for 1 min with 0.22 μm of filtered 2% uranyl acetate (Ladd Research, #23620) in double-distilled water. The grid was blotted for 3 s using Whatman Grade 1 filter paper (Whatman #1001-085) and air-dried on inverted tweezers. Images were captured with a FEI Tecnai Spirit G2 BioTWIN operated at 80 KeV and equipped with a bottom-mounted 4kx4k FEI Eagle camera.
