# Supplementary material for: Structure of the Chimalliviridae bacteriophage Goslar reveals host recognition and infection machinery
Source: Sci Adv. 2026 Nov 6;12(45):eaei4524. doi: 10.1126/sciadv.aei4524 (PMC13742148; doi:10.1126/sciadv.aei4524)
Supplement: Supplementary file 1 [file sciadv.aei4524_sm.pdf]

Science Advances
AAAS

Supplementary Materials for

Structure of the *Chimalliviridae* bacteriophage Goslar reveals host recognition and infection machinery

Dwaipayan Basu *et al.*

Corresponding author: Joe Pogliano, jpogliano@ucsd.edu; Kevin D. Corbett, kcorbett@ucsd.edu

Sci. Adv. **12**, eaei4524 (2026)
DOI: 10.1126/sciadv.aei4524

The PDF file includes:

Figs. S1 to S21
Table S1
Legends for data S1 to S3

Other Supplementary Material for this manuscript includes the following:

Data S1 to S3

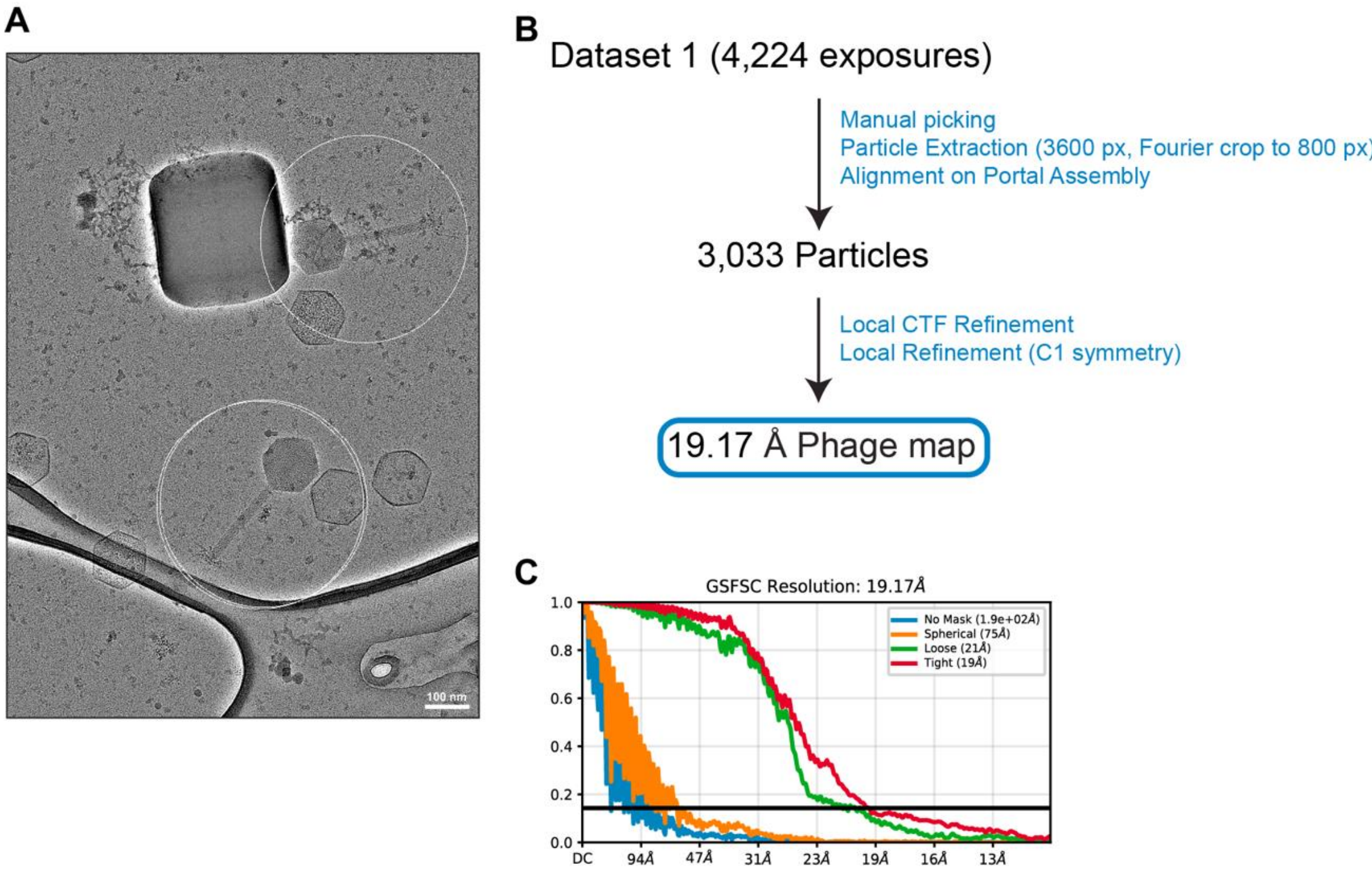

Figure S1. CryoEM workflow for asymmetric reconstruction of Goslar phage. (A) Example cryoEM micrograph from dataset 1 (collected in super-resolution mode with pixel size of 1.3 Å), with white circles indicating manually-picked intact Goslar phages. Also visible are several empty capsids without tails (see **Fig. S4**). Scale bar: 100 nm. **(B)** CryoEM workflow for structure determination of intact Goslar phages. See **Data S1** for data collection and refinement statistics. **(C)** Gold-standard Fourier Shell Correlation (GSFSC) curves for the 19.17 Å resolution icosahedral map.

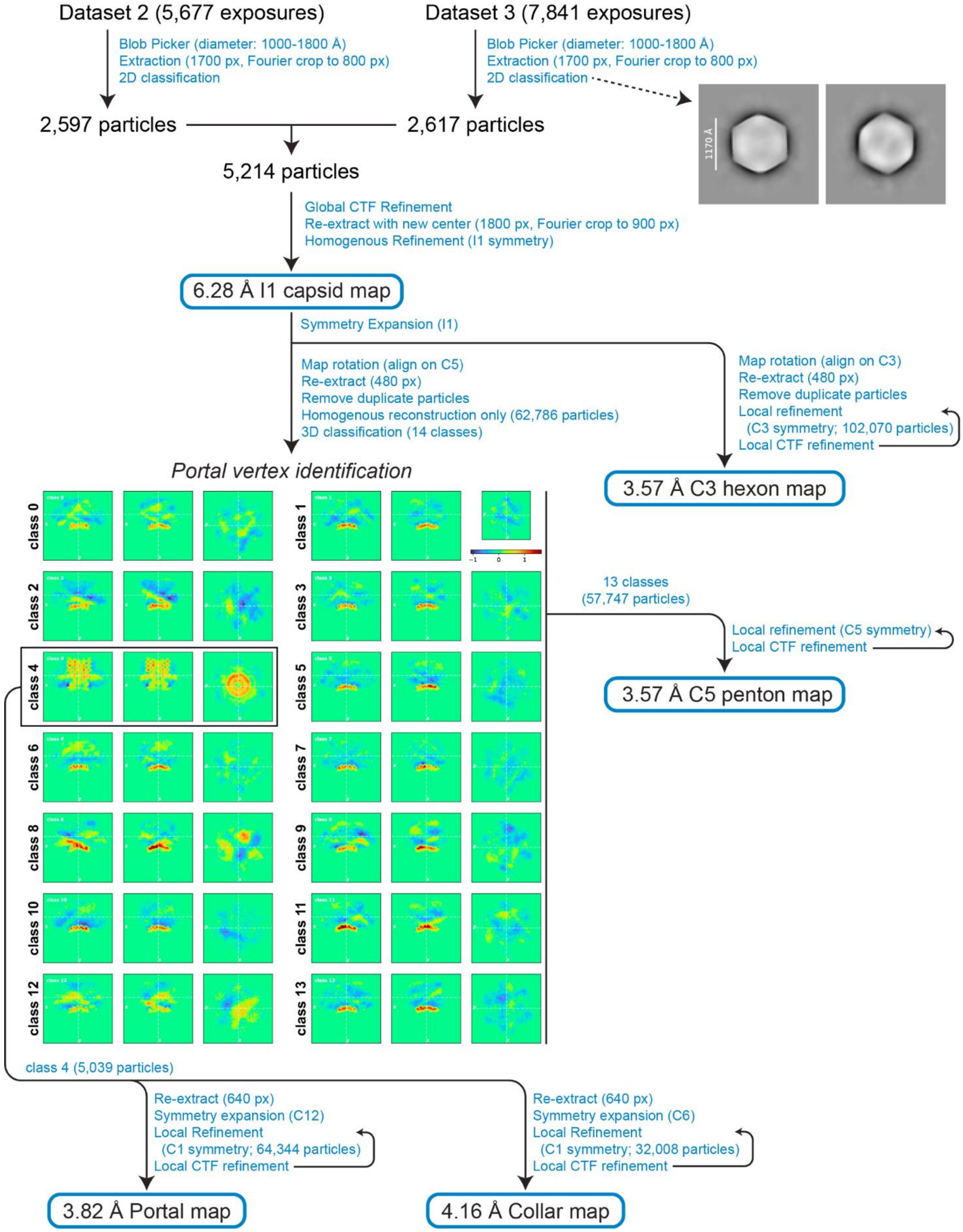

Figure S2. CryoEM workflow for Goslar capsid, portal, and neck reconstructions. CryoEM workflow for structure determination of Goslar capsid, portal, and neck. From two datasets,

capsid particles were picked using the blob picker, extracted, then combined for an initial 3D reconstruction with icosahedral symmetry. Particles were then symmetry-expanded, maps were rotated to align on either the five-fold (C5) or three-fold (C3) symmetry axis of the capsid, then re-extracted. For particles aligned on the C3 axis, iterative local refinement and local CTF estimation yielded a 3.57 Å resolution C3 "hexon" map. For particles aligned on the C5 axis, homogeneous reconstruction and 3D classification identified the vertex of each particle associated with the portal. These particles were subjected to iterative local refinement and local CTF estimation with C12 symmetry to generate a 3.82 Å resolution portal/upper neck map, or with C6 symmetry to generate a 4.16 Å resolution lower neck map. All vertices not associated with a portal were combined and refined with C5 symmetry to generate a 3.57 Å resolution C5 "penton" map. See **Data S2** for data collection and refinement statistics.

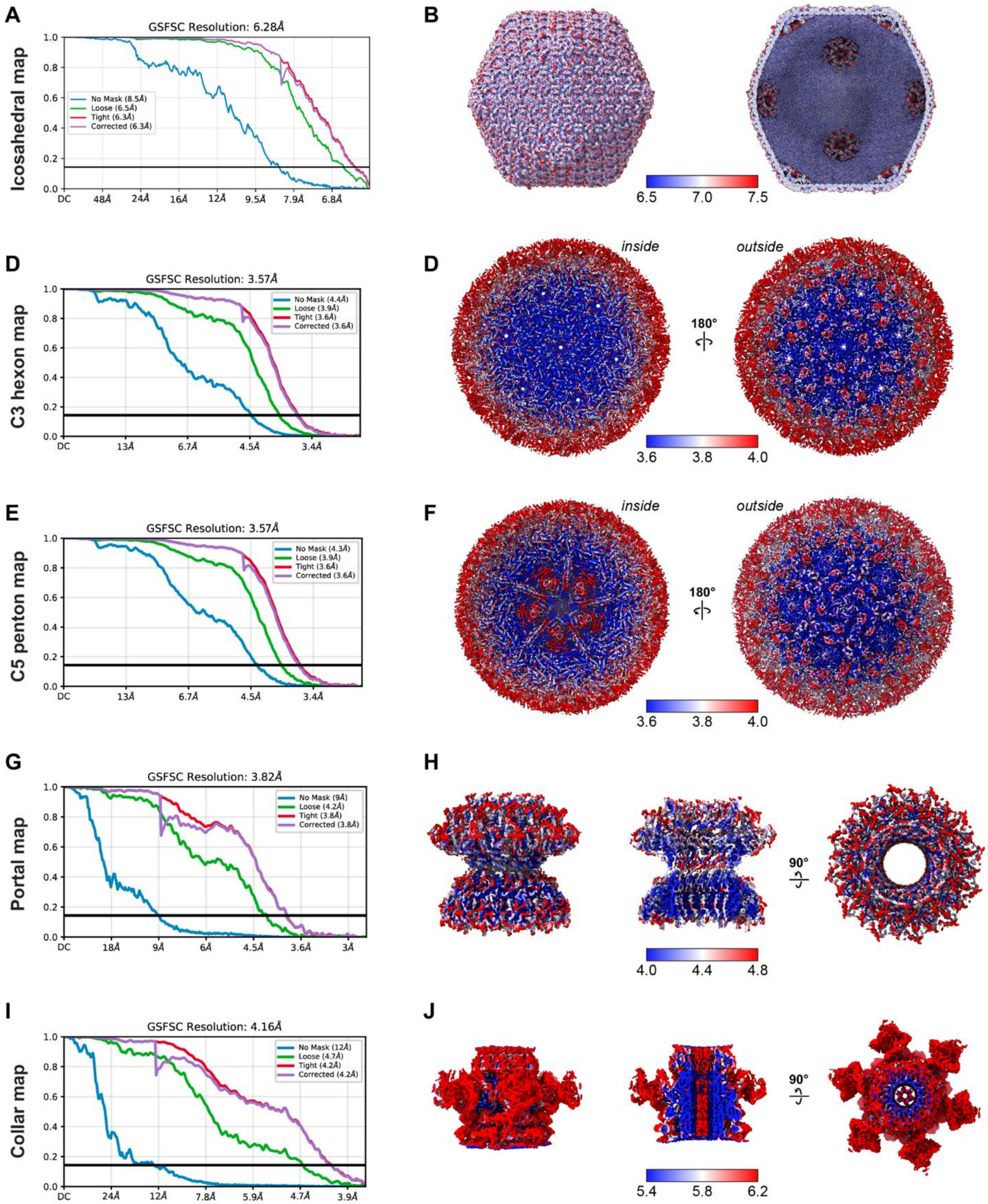

Figure S3. Validation of Goslar capsid, portal, and neck reconstructions. (A) Gold-standard Fourier Shell Correlation (GSFSC) curves for the 6.28 Å resolution icosahedral map. **(B)** Exterior (left) and cutaway (right) views of the icosahedral map, colored by local resolution. **(C)** GSFSC curves for the 3.57 Å resolution C3 "hexon" map. **(D)** Inside (left) and outside (right) views of the C3 "hexon" map, colored by local resolution. **(E)** GSFSC curves for the 3.57 Å

resolution C5 “penton” map. **(F)** Inside (left) and outside (right) views of the C5 “penton” map, colored by local resolution. **(G)** GSFSC curves for the 3.82 Å resolution C12 portal map. **(H)** Side view (left), cutaway view (center), and top view (right) of the C12 portal/upper neck map, colored by local resolution. **(I)** GSFSC curves for the 4.16 Å resolution C6 lower neck map. **(J)** Side view (left), cutaway view (center), and top view (right) of the C6 lower neck map, colored by local resolution.

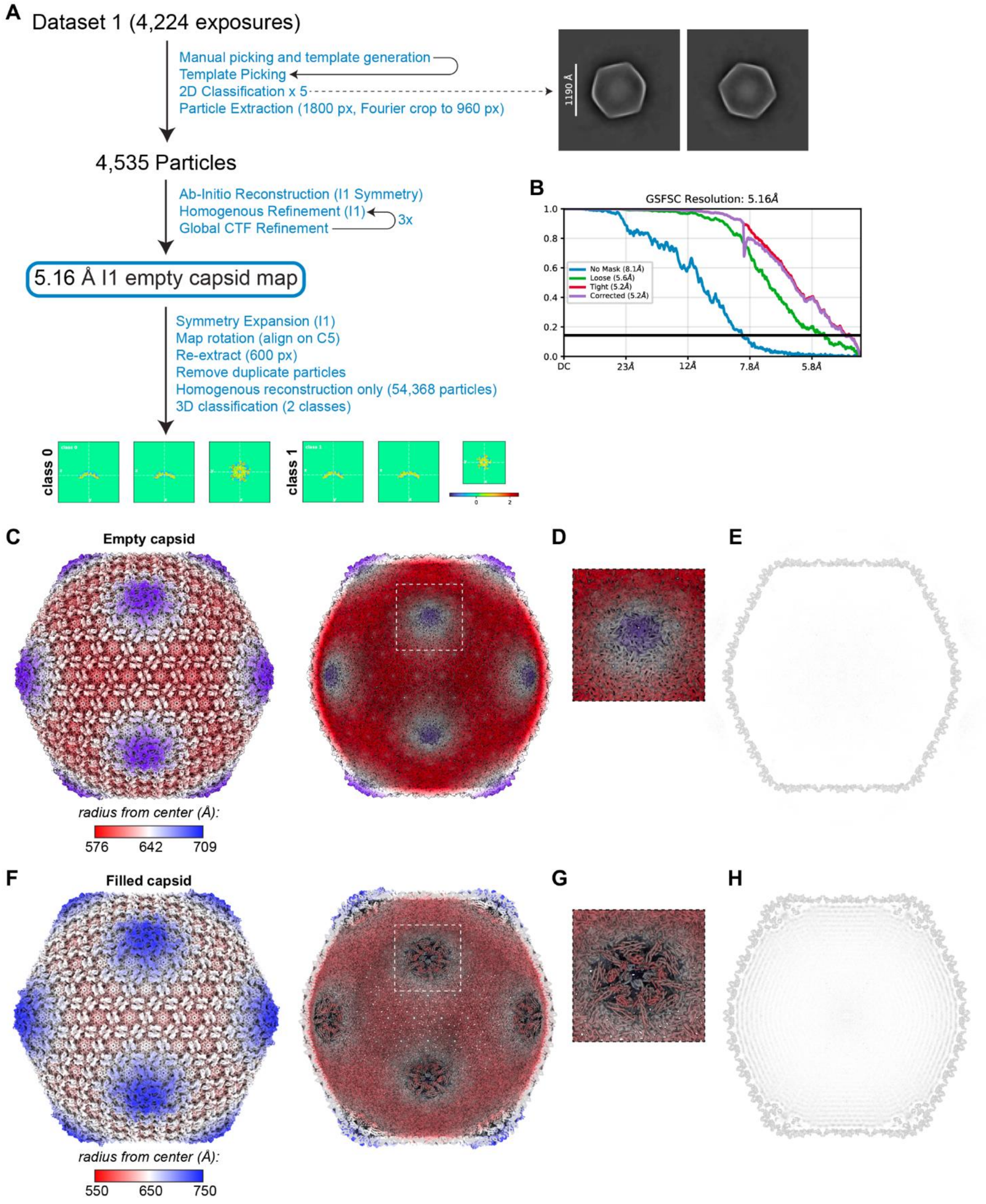

Figure S4. CryoEM workflow for empty capsid reconstruction. (A) CryoEM workflow for structure determination of Goslar empty capsids. 3D classification (results of a trial with 2 classes is shown; trials with up to 10 classes showed consistent results) did not reveal a portal vertex. See **Data S1** for data collection and refinement statistics. **(B)** Gold-standard Fourier Shell

Correlation (GSFSC) curves for the 5.16 Å resolution icosahedral map. **(C)** Exterior and cutaway views of the icosahedral map for empty capsids, colored by radius from the center. **(D)** Closeup view of the inside of one C5 vertex in the empty capsid, showing the lack of a vertex binding complex. **(E)** Cross-section of the icosahedral map for empty capsids. **(F)** Exterior and cutaway views of the icosahedral map for DNA-filled capsids (see **Figs. S2-S3**), colored by radius from the center. **(G)** Closeup view of the inside of one C5 vertex in the DNA-filled capsid, showing the vertex binding complex. **(H)** Cross-section of the icosahedral map for the DNA-filled capsid, showing the vertex-binding complex and layers of density corresponding to DNA.

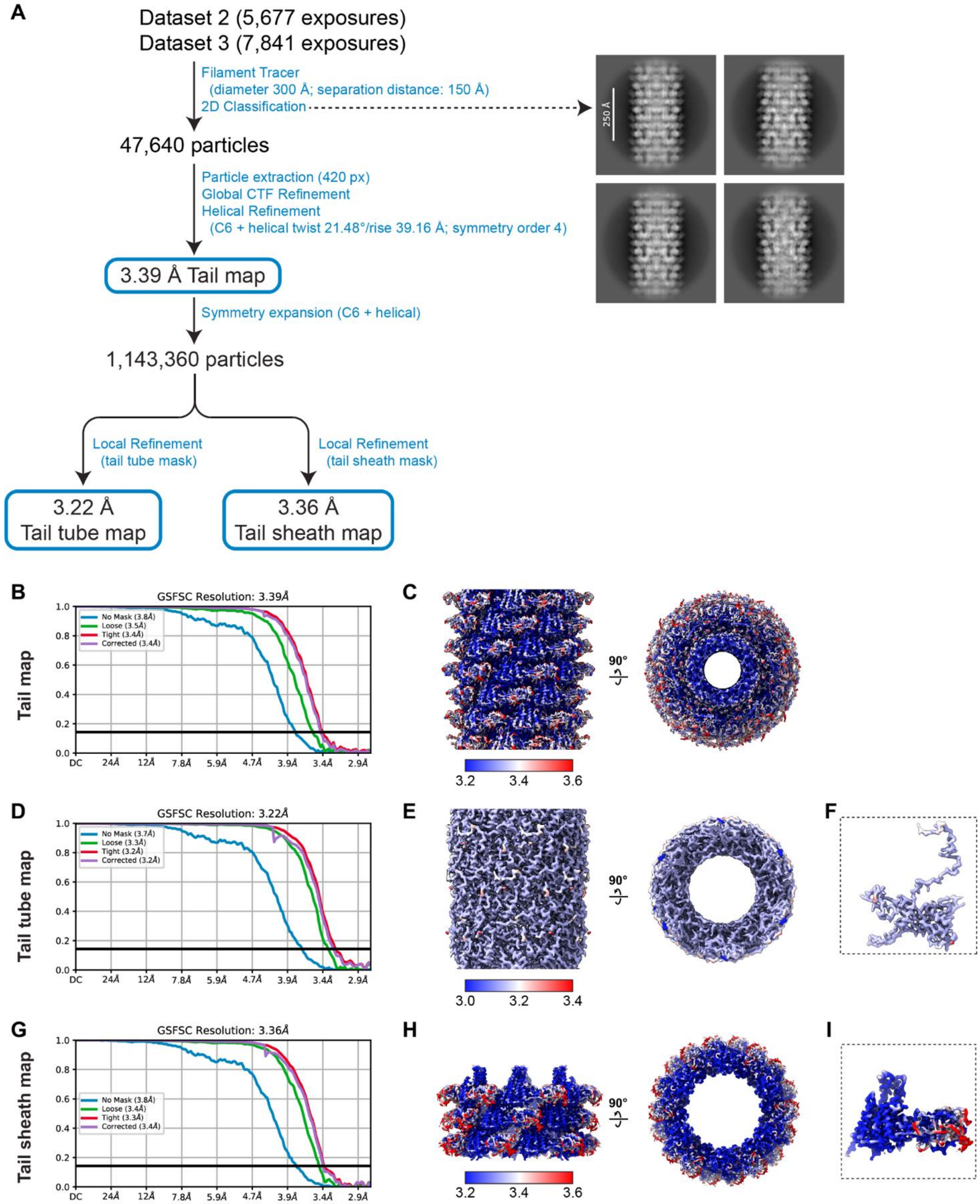

Figure S5. CryoEM workflow for Goslar tail reconstructions. (A) From two datasets, the cryoSPARC Filament Tracer was used to pick particles, followed by 2D classification to yield a 47,640 particle set. Helical refinement with C6 symmetry yielded a 3.39 Å resolution tail map.

Symmetry expansion and local refinement with masks yielded a 3.22 Å resolution tail tube map, and a 3.36 Å resolution tail sheath map. See **Data S2** for data collection and refinement statistics. **(B)** GSFSC curves for the 3.39 Å resolution tail map. **(C)** Side and top views of the 3.39 Å resolution tail map, colored by local resolution. **(D)** GSFSC curves for the 3.22 Å resolution tail tube map. **(E)** Side and top views of the 3.22 Å resolution tail map, colored by local resolution. **(F)** View of a single tail tube protein protomer, colored by local resolution. **(G)** GSFSC curves for the 3.36 Å resolution tail sheath map. **(H)** Side and top views of the 3.36 Å resolution tail sheath map, colored by local resolution. **(I)** View of a single tail sheath protein protomer, colored by local resolution.

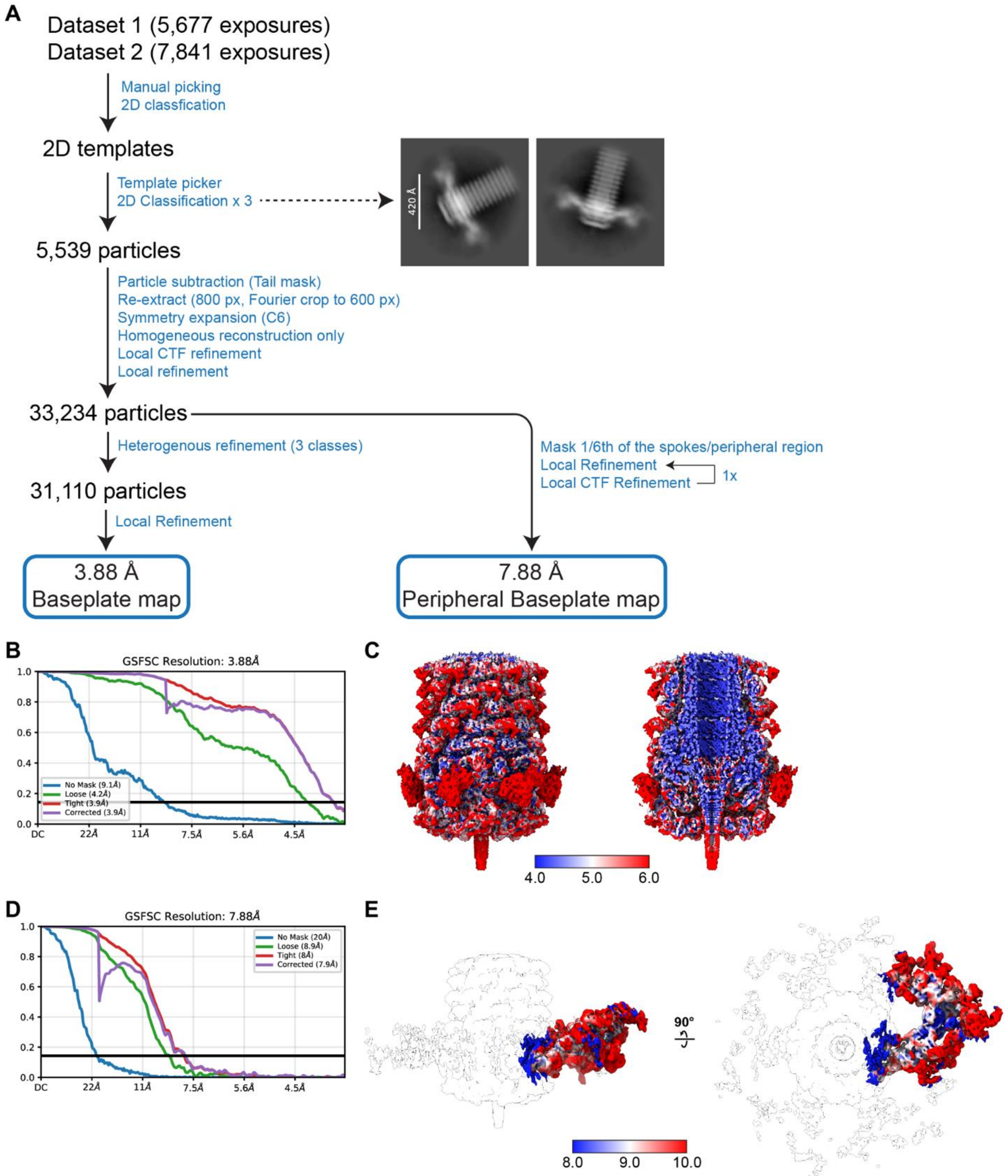

Figure S6. CryoEM workflow for Goslar baseplate reconstruction. (A) From two datasets, manually-picked particles were used to generate templates for template-based particle picking. After 2D classification, a 5,539 particle set was used for initial reconstruction and refinement with C6 symmetry. Symmetry expansion followed by local refinement resulted in a final map at 3.88 Å resolution. To better resolve the spokes and peripheral region, a manual mask around 1/6th of that region was used for a second local refinement, resulting in a 7.88 Å resolution map.

See **Data S2** for data collection and refinement statistics. **(B)** GSFSC curves for the 3.88 Å resolution baseplate map. **(C)** Side and cutaway views of the 3.88 Å resolution baseplate map, colored by local resolution. **(D)** GSFSC curves for the 7.88 Å resolution spoke/peripheral baseplate map. **(E)** Side and top views of the 7.88 Å resolution spoke/peripheral baseplate map, colored by local resolution.

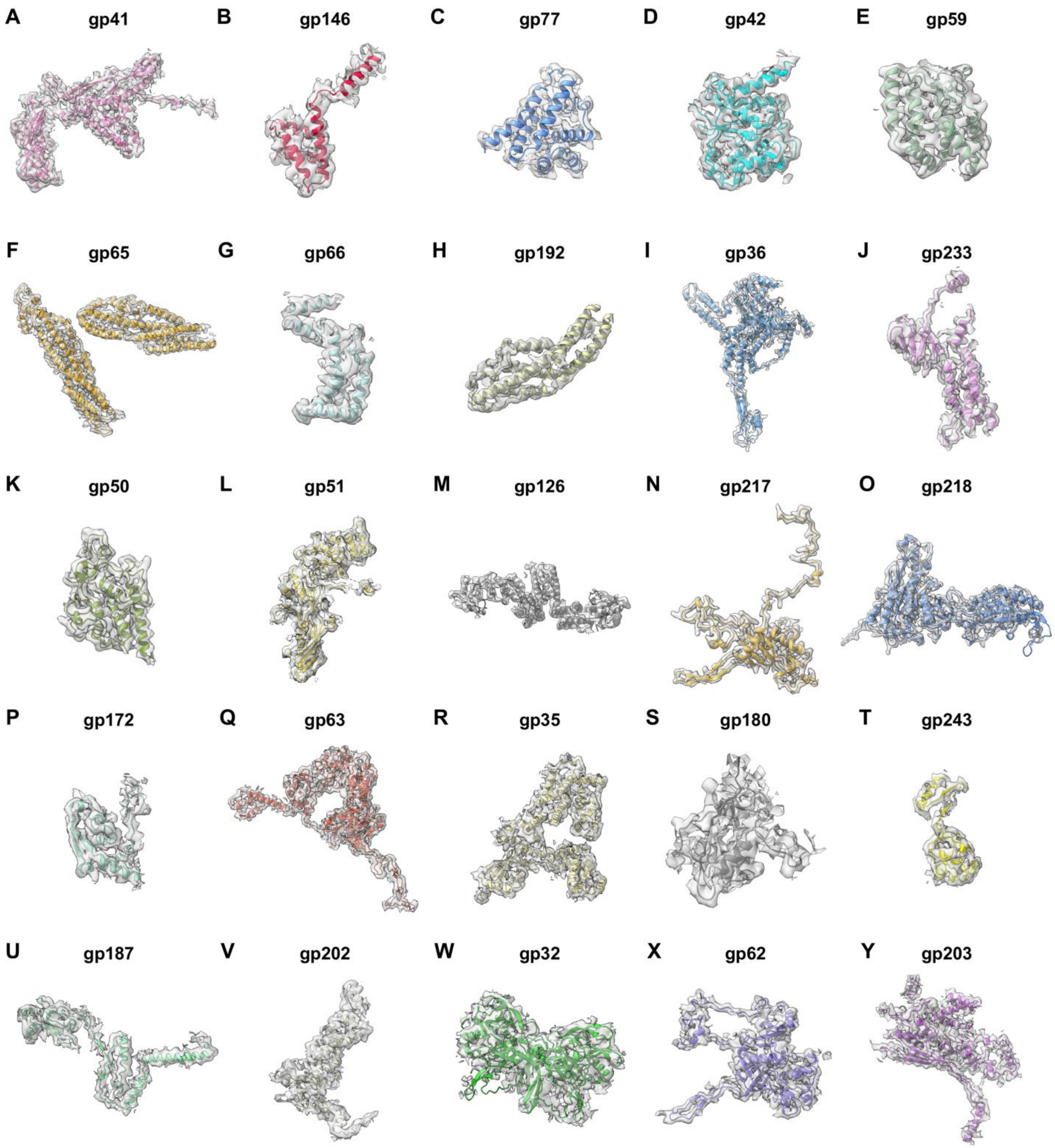

Figure S7. Example models and cryoEM density for 24 manually-refined proteins. (A-Y) Cartoon models and corresponding cryoEM density for one protomer of each of 24 structural proteins that were manually modeled and subjected to atomic positional and atomic displacement parameter (ADP) refinement.

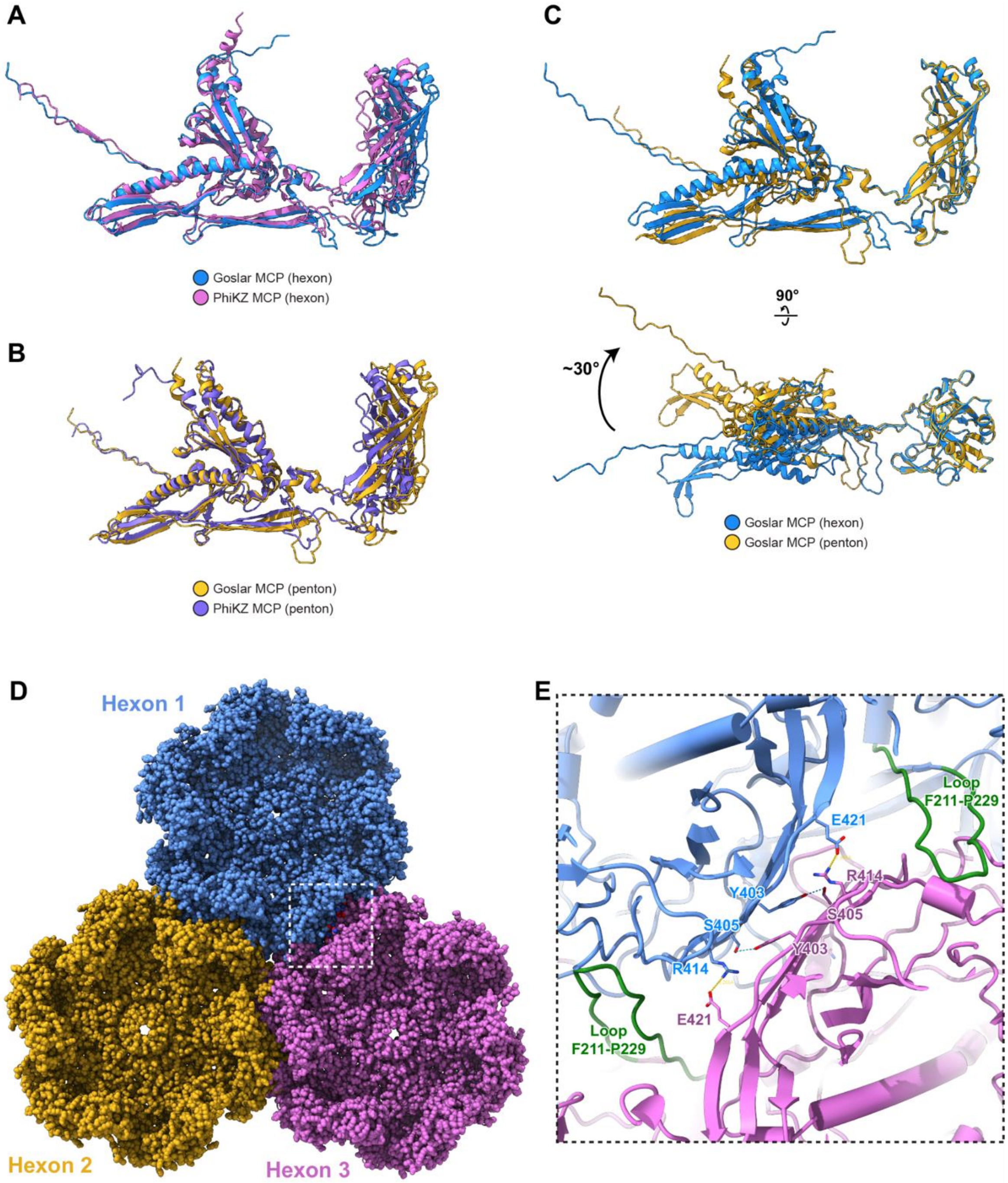

Figure S8. Structure and packing of the Goslar major capsid protein. (A) Overlay of the Goslar major capsid protein in the hexon (gp41; blue) with that of PhiKZ (purple; PDB ID 8Y6V) *(41)*. **(B)** Overlay of Goslar gp41 in the penton (yellow) with that of PhiKZ. **(C)** Two views of Goslar gp41 in the hexon (blue) and penton (yellow), showing rotation of the I domain relative to the rest of the protein. **(D)** View of three gp41 hexons packing against one another in the capsid. **(E)** Closeup view of the interactions between gp41 protomers in neighboring hexons.

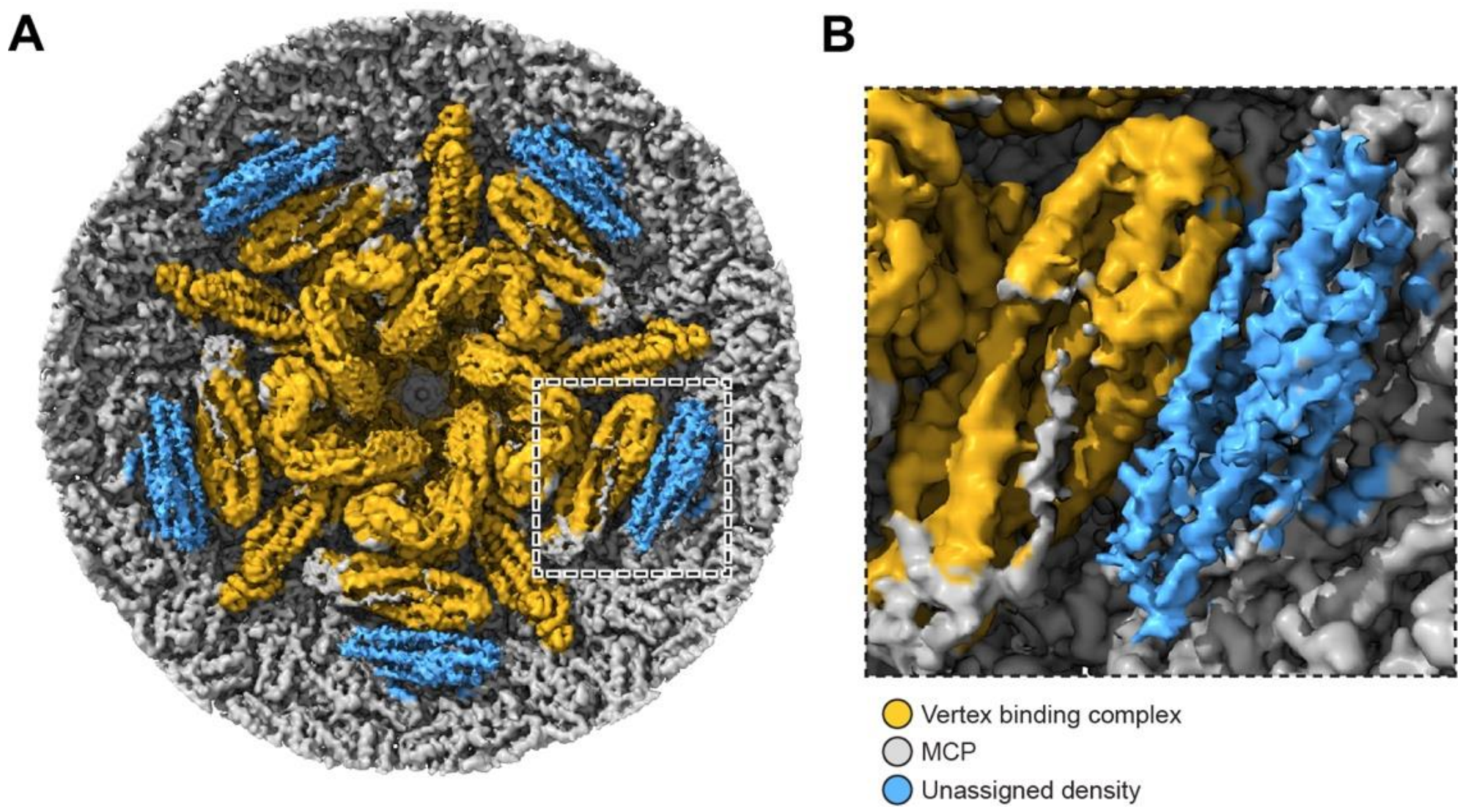

Figure S9. Unassigned density near the vertex binding complex. (A) CryoEM density for the vertex binding complex at low contour level, viewed from inside the Goslar capsid. Density corresponding to the vertex binding complex is colored yellow; density corresponding to the capsid (major capsid protein; MCP) is gray), and unassigned density is blue. **(B)** Closeup view of one unassigned density (blue). The neighboring yellow density corresponds to gp65.

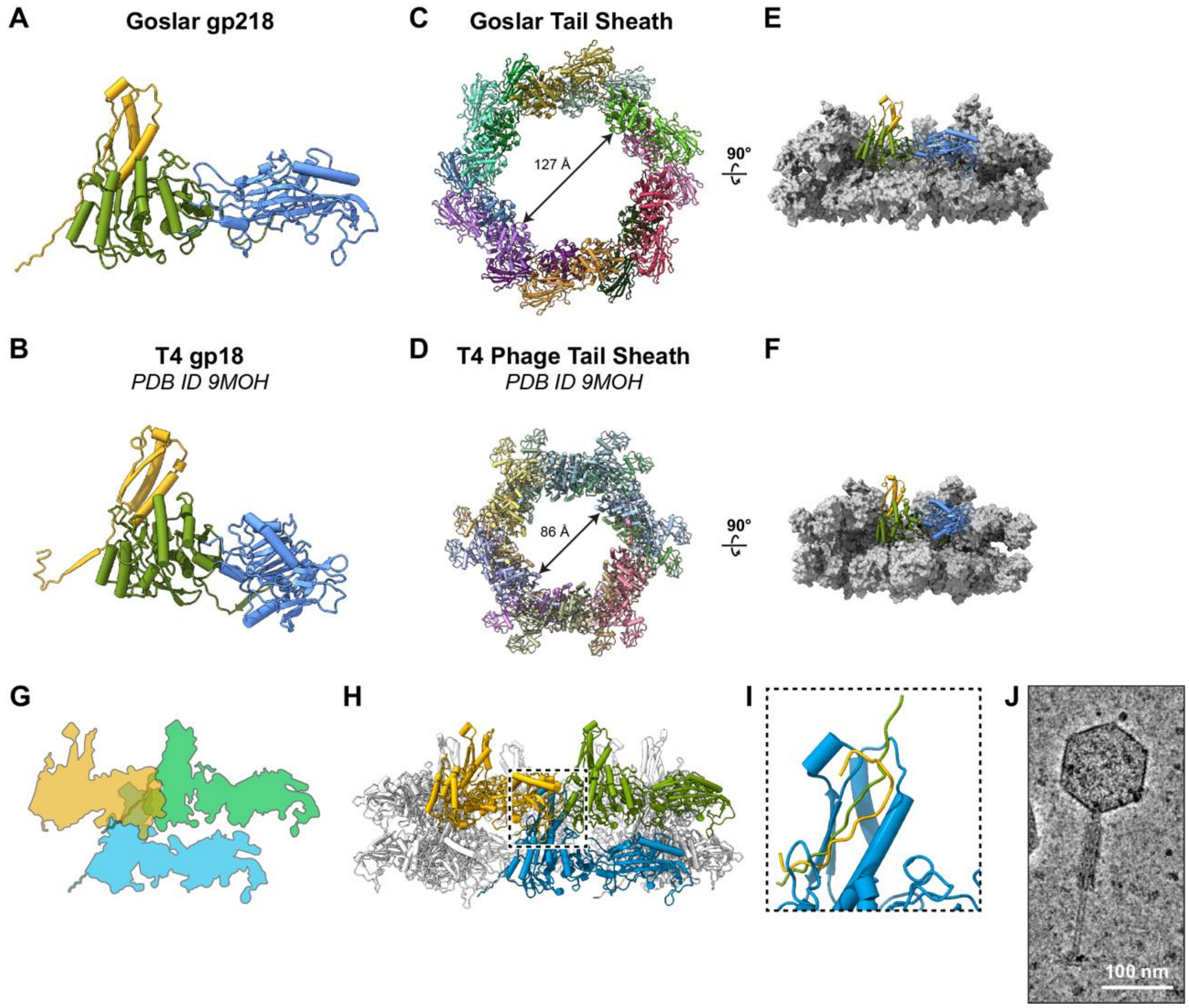

Figure S10. Goslar tail sheath architecture and conservation. (A) Cartoon view of the Goslar tail sheath protein gp218, with central domain green, outer sheath domain blue, and C-terminal domain and extension yellow. **(B)** Cartoon view of the T4 tail sheath protein gp18 (PDB ID 9MOH) *(46)*, with domains colored as in panel (A). **(C)** Top-down view of the Goslar tail sheath, with inner diameter noted. **(D)** Top-down view of the T4 tail sheath (PDB ID 9MOH) *(46)*, with inner diameter noted. **(E)** Side view of the Goslar tail sheath, with one protomer shown as cartoon and colored as in panel (A). **(F)** Side view of the T4 tail sheath, with one protomer colored as in panel (B). **(G)** Outline schematic showing the positioning of three interacting protomers of the tail sheath protein gp218. **(H)** Cartoon view of three interacting tail sheath protomers in the context of the full tail sheath assembly (two layers shown). **(I)** Closeup of the critical interaction between the C-terminal domain of one tail sheath protomer (blue) with two other protomers (yellow and green) in the layer above. **(J)** Electron micrograph of a Goslar virion with a contracted tail sheath.

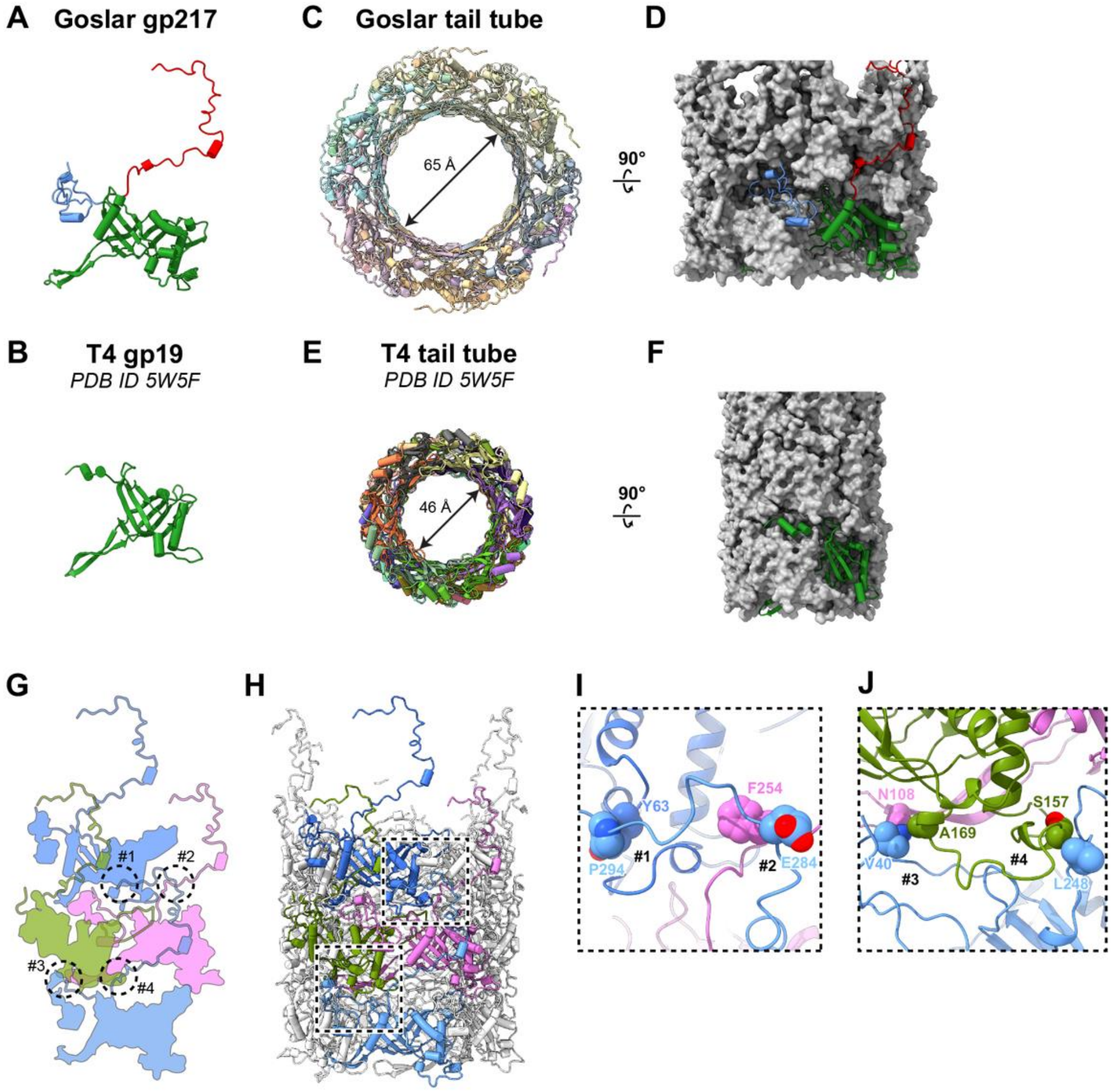

Figure S11. Goslar tail tube architecture and conservation. (A) Cartoon view of the Goslar tail tube protein gp217, with central domain green, N-terminal domain blue, and C-terminal extension red. **(B)** Cartoon view of the T4 tail tube protein gp19 (PDB ID 5W5F) *(48)*, with domains colored as in panel (A). **(C)** Top-down view of the Goslar tail tube, with inner diameter noted. **(D)** Top-down view of the T4 tail tube (PDB ID 5W5F) *(48)*, with inner diameter noted. **(E)** Side view of the Goslar tail tube, with one protomer shown as cartoon and colored as in panel (A). **(F)** Side view of the T4 tail tube, with one protomer colored as in panel (B). **(G)** Outline schematic showing the positioning of four interacting protomers of the tail tube protein gp217. Key interactions are marked #1 through #4. **(H)** Cartoon view of the four interacting tail tube protomers in the contect of the full tail tube assembly (three layers shown). **(I)** Closeup of interactions #1 and #2. **(J)** Closeup of interactions #3 and #4.

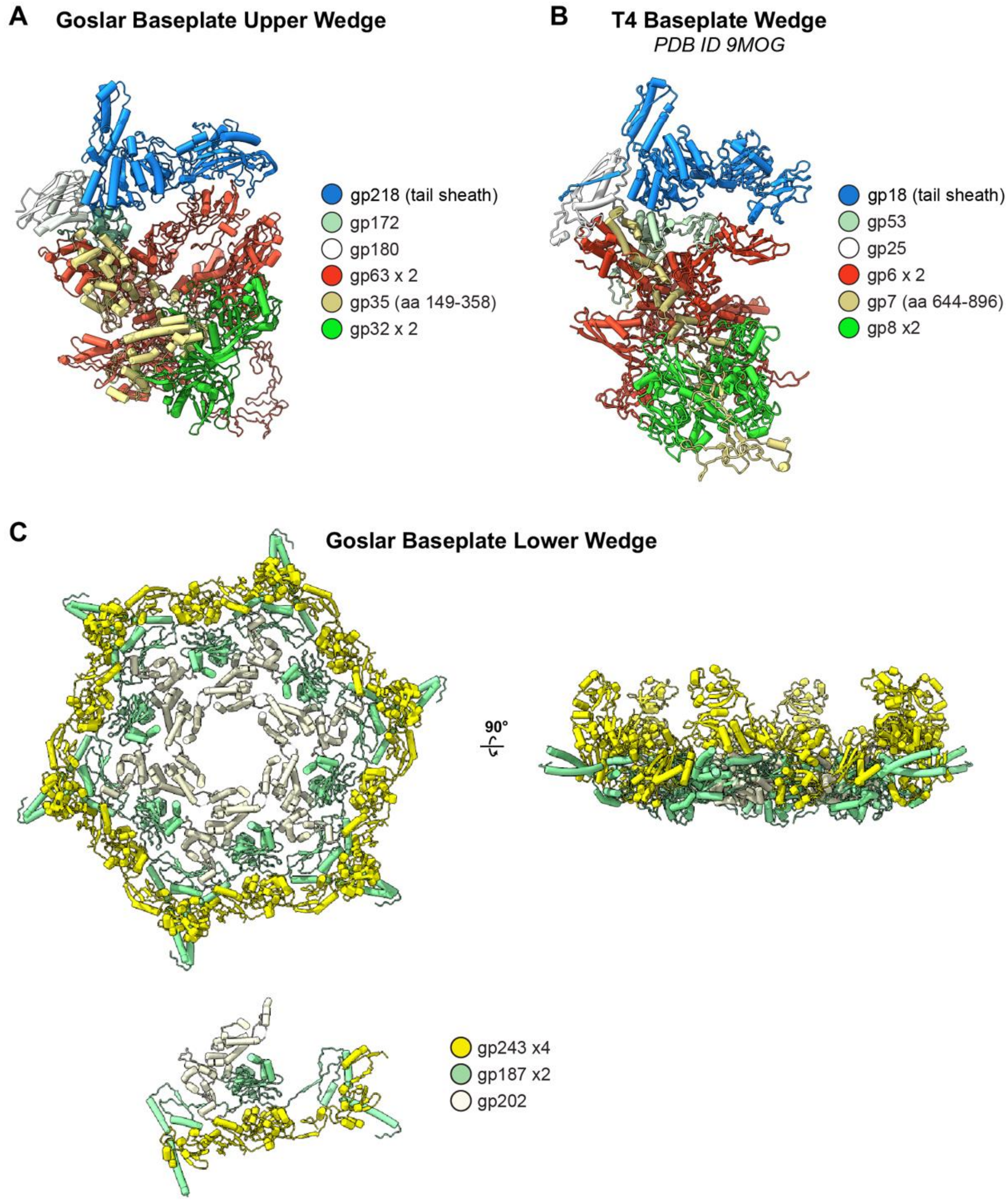

Figure S12. Goslar baseplate wedge architecture and conservation. (A) Cartoon view of one upper wedge of the Goslar baseplate, with proteins colored as indicated in the key. Two copies of gp63 and gp32 are shown, and only residues 149-358 for gp35 (equivalent to residues 644-896 of T4 gp7) are shown. **(B)** Cartoon view of one wedge of the T4 baseplate (from PDB ID 9MOG) *(46)*, with proteins colored as indicated in the key. Homologous proteins in panels (A) and (B) are colored consistently, and the views of each are aligned on their respective tail sheath proteins. Two copies of gp6 and gp8 are shown, and only residues 644-896 for gp7 (equivalent to residues 149-358 of Goslar gp35) are shown. **(C)** *Top:* Two views of the Goslar baseplate lower wedge ring ,with proteins colored as indicated. *Bottom:* Top-down view of one lower wedge.

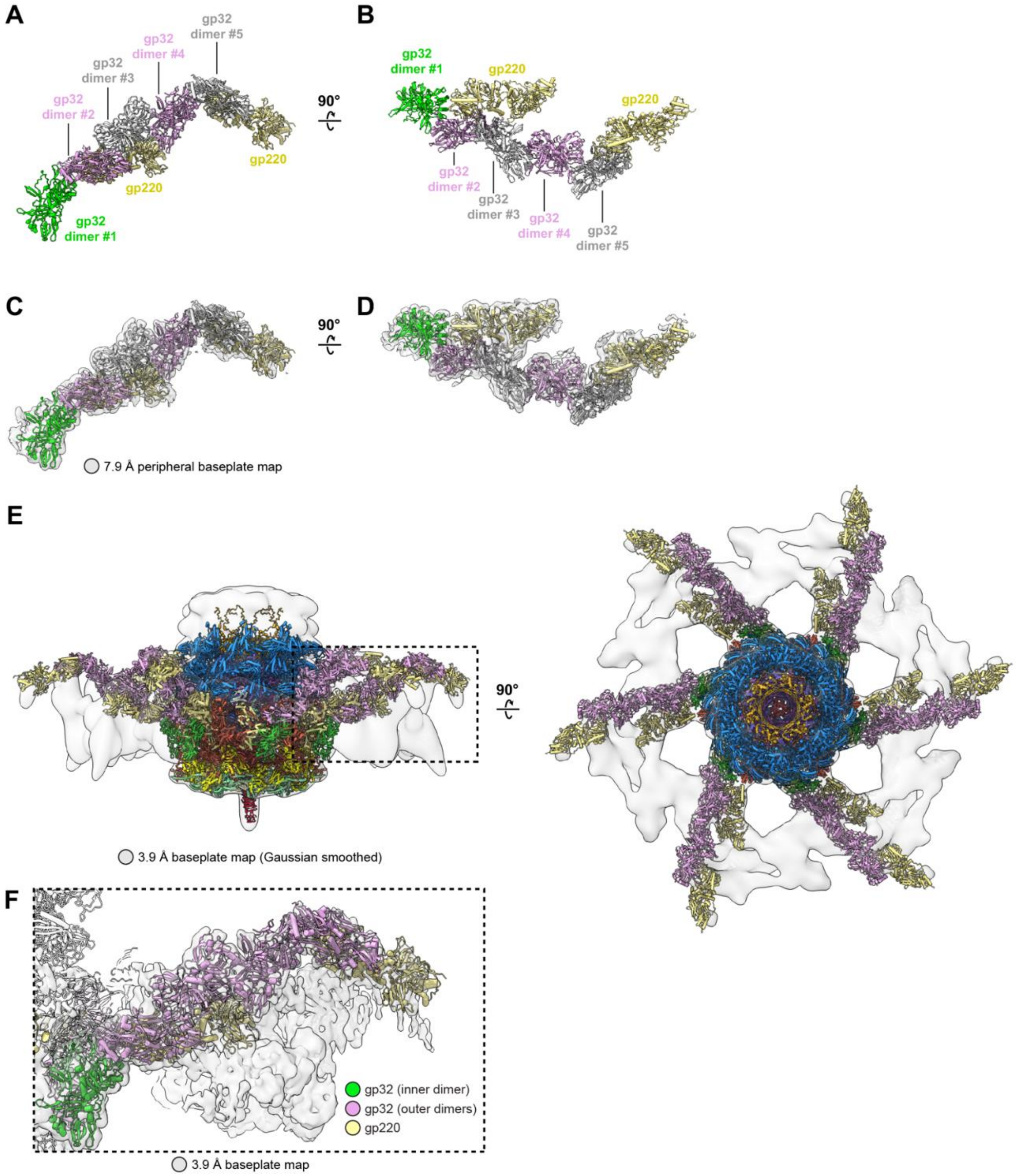

Figure S13. Spoke and peripheral baseplate architecture. (A) Side view of one spoke, with five dimers of gp32 and two copies of gp220 labeled. **(B)** Top-down view of one spoke. **(C)** Side view of one spoke, with cryoEM density from the 7.9 Å resolution peripheral baseplate map shown. **(D)** Top-down view of one spoke, with cryoEM density from the 7.9 Å resolution peripheral baseplate map shown. **(E)** Side and top-down views of the entire baseplate molecular model, with atomic models colored by protein as in **Fig. 6B-D**, and Gaussian-smoothed cryoEM density from the 3.9 Å resolution baseplate map shown in gray. **(F)** Closeup of one spoke, with five dimers of gp32 and two copies of gp220 shown as cartoons, and cryoEM density from the 3.9 Å resolution baseplate map (not smoothed) shown in gray.

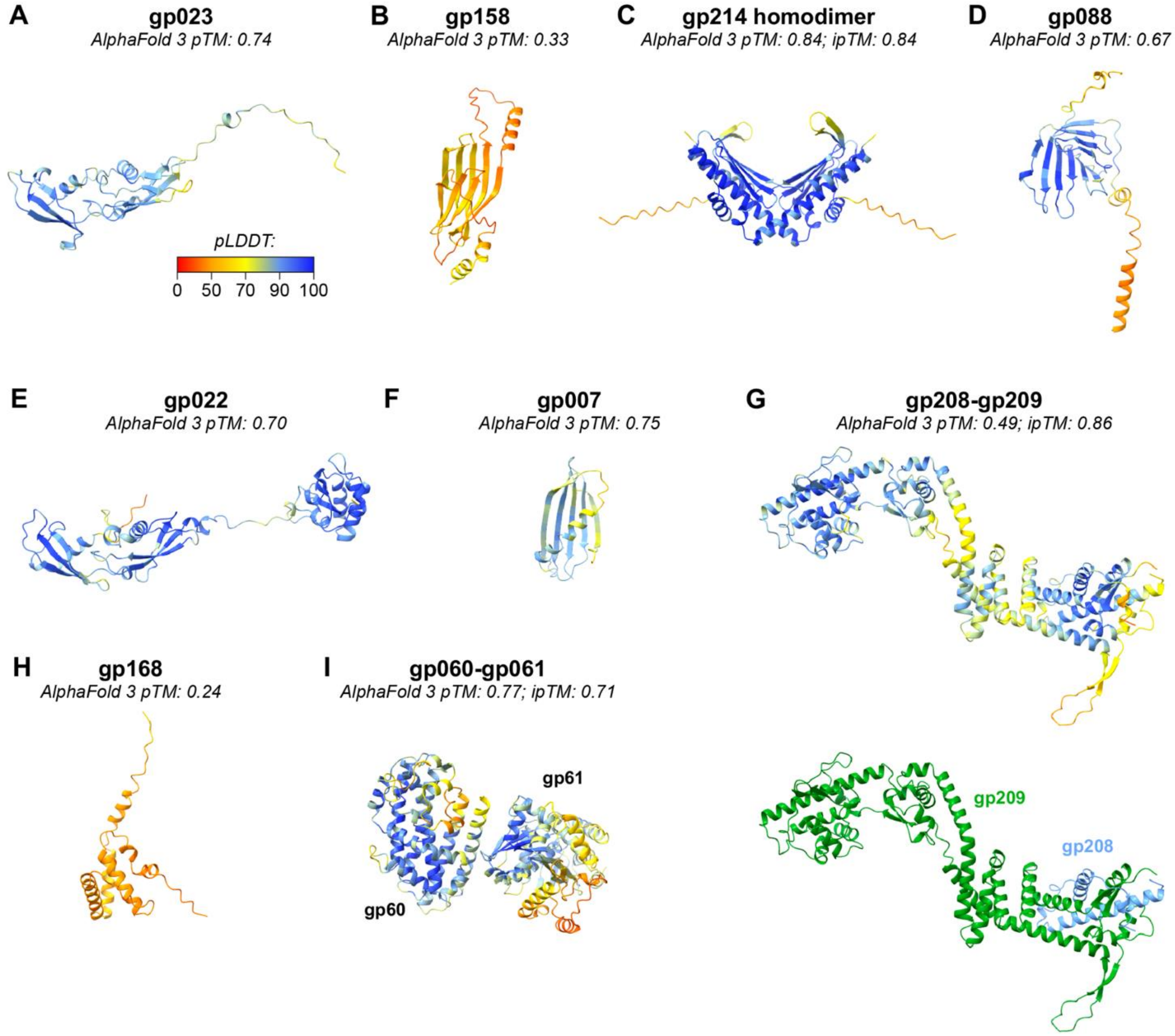

Figure S14. AlphaFold-predicted structures of high-copy Goslar virion proteins. (A) Alphafold 3 prediction of Goslar gp023, colored by local confidence (pLDDT). **(B)** Alphafold 3 prediction of Goslar gp158. **(C)** Alphafold 3 prediction of a homodimer of Goslar gp214. **(D)** Alphafold 3 prediction of Goslar gp088. **(E)** Alphafold 3 prediction of Goslar gp022. **(F)** Alphafold 3 prediction of Goslar gp007. **(G)** Alphafold 3 prediction of a heterodimeric Goslar gp208-gp209 complex. Top: colored by pLDDT. Bottom: colored by protein with gp208 blue and gp209 green. **(H)** Alphafold 3 prediction of Goslar gp168. **(I)** Alphafold 3 prediction of a heterodimeric Goslar gp060-gp061 complex, colored by pLDDT.

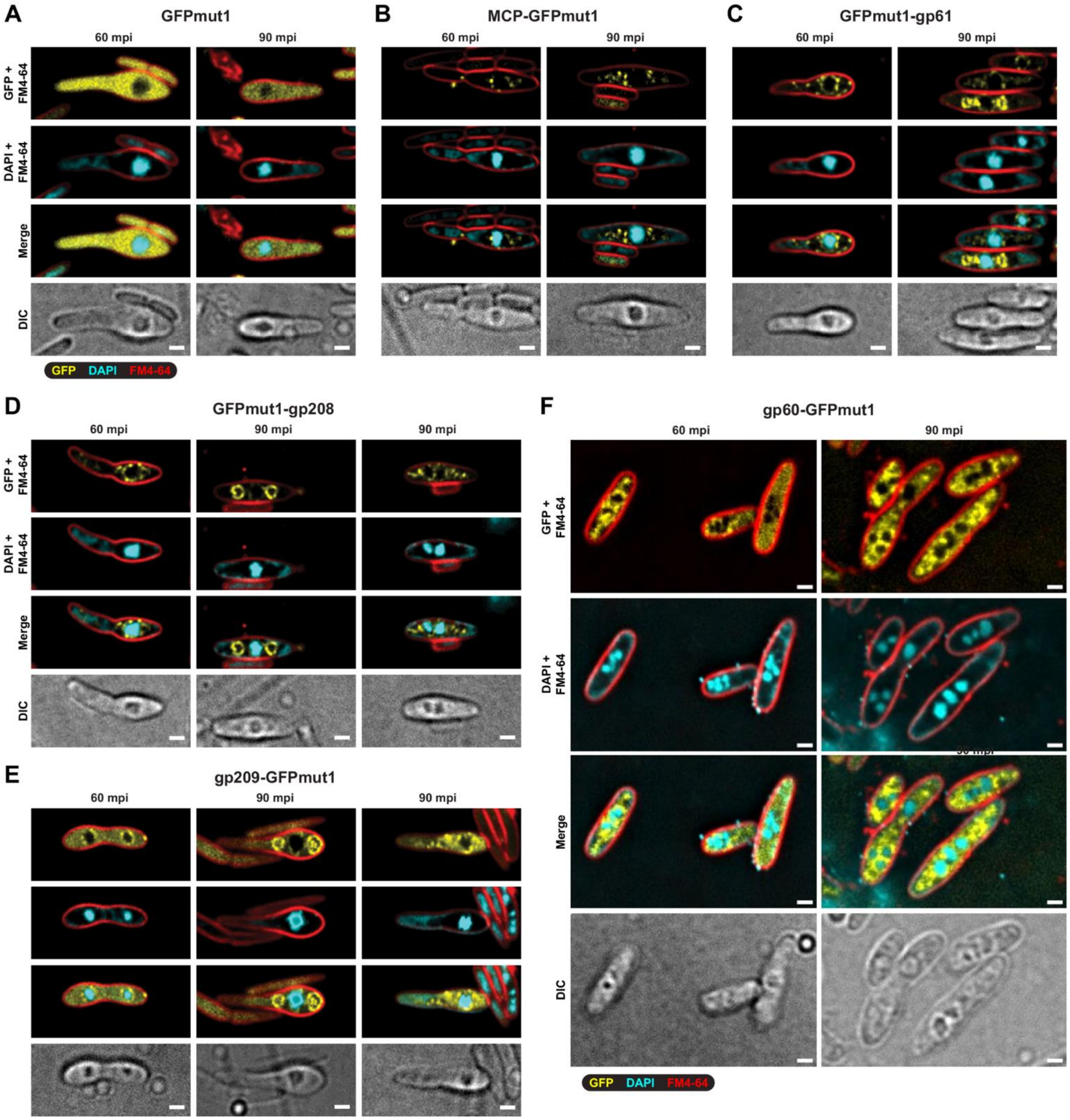

Figure S15. Localization of high-copy virion-associated proteins in Goslar-infected cells. **(A)** Subcellular localization of GFPmut1 in Goslar-infected *E. coli* MC1000 cells, 60 or 90 minutes post infection (mpi). GFP is shown in yellow; DAPI (DNA) in cyan; and FM4-64 (membranes) in red. Differential interference contrast (DIC) imaging is shown at bottom. Scale bar = 1 µm. **(B)** Localization of Goslar gp41 (major capsid protein; MCP) tagged with GFPmut1 at the C-terminus. **(C)** Localization of Goslar gp61 tagged with GFPmut1 at the N-terminus. **(D)** Localization of Goslar gp208 tagged with GFPmut1 at the N-terminus. Two cells are shown at 90 mpi. **(E)** Localization of Goslar gp209 tagged with GFPmut1 at the C-terminus. The cell shown at 60 mpi is double-infected, and shows two phage nuclei. Two cells are shown at 90 mpi. **(F)** Localization of Goslar gp60 tagged with GFPmut1 at the C-terminus.

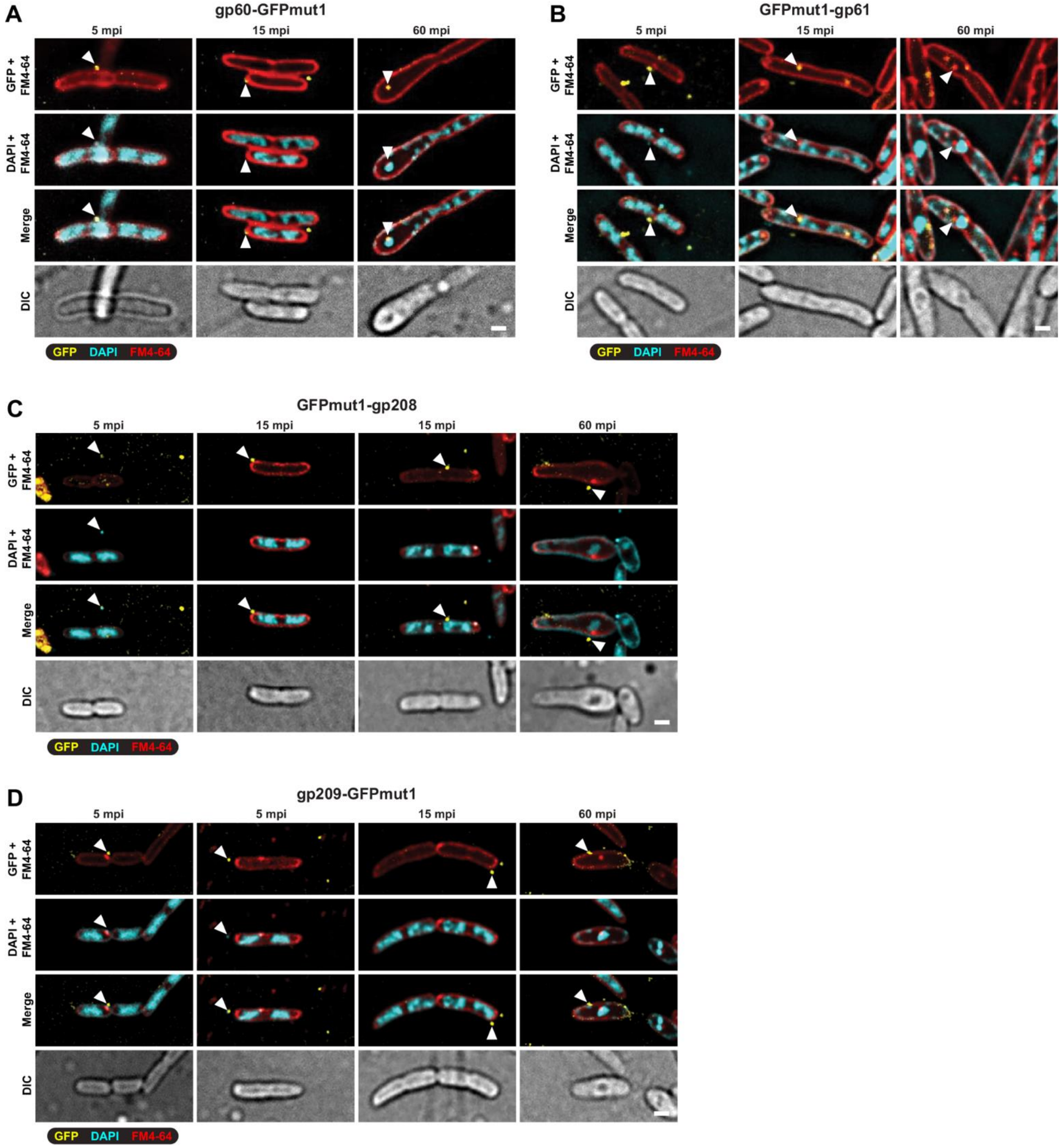

Figure S16. Virion packaging and host injection of Goslar virion-associated proteins. (A) Localization of gp60-GFPmut1 packaged into virions and infecting *E. coli* MC1000 cells, at 5, 15, or 60 minutes post infection (mpi). GFP is shown in yellow; DAPI (DNA) in cyan; and FM4-64 (membranes) in red. Differential interference contrast (DIC) imaging is shown at bottom. Scale bar = 1 µm. **(B)** Localization of GFPmut1-gp61 packaged into virions. **(C)** Localization of GFPmut1-gp208 packaged into virions. **(D)** Localization of gp209-GFPmut1 packaged into virions.

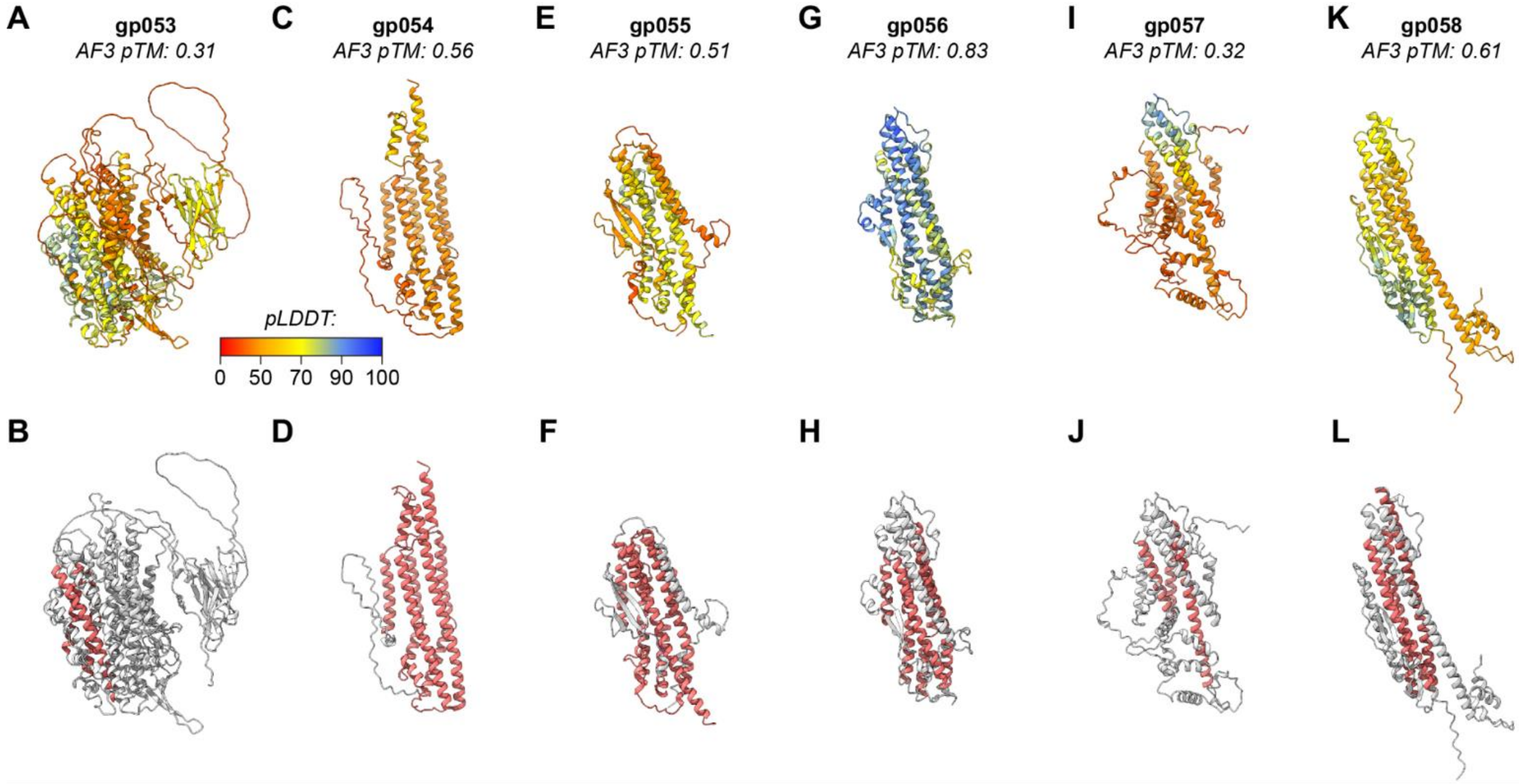

Figure S17. Predicted structures of Goslar PF12699 proteins. (A) Alphafold 3 prediction of Goslar gp053, colored by local confidence (pLDDT). **(B)** Alphafold 3 prediction of Goslar gp053, with secondary structure elements corresponding to the core PF12699 fold shown in red. **(C)** Alphafold 3 prediction of Goslar gp054, colored by local confidence (pLDDT). **(D)** Alphafold 3 prediction of Goslar gp054, with secondary structure elements corresponding to the core PF12699 fold shown in red. **(E)** Alphafold 3 prediction of Goslar gp055, colored by local confidence (pLDDT). **(F)** Alphafold 3 prediction of Goslar gp055, with secondary structure elements corresponding to the core PF12699 fold shown in red. **(G)** Alphafold 3 prediction of Goslar gp056, colored by local confidence (pLDDT). **(H)** Alphafold 3 prediction of Goslar gp056, with secondary structure elements corresponding to the core PF12699 fold shown in red. **(I)** Alphafold 3 prediction of Goslar gp057, colored by local confidence (pLDDT). **(J)** Alphafold 3 prediction of Goslar gp057, with secondary structure elements corresponding to the core PF12699 fold shown in red. **(K)** Alphafold 3 prediction of Goslar gp058, colored by local confidence (pLDDT). **(L)** Alphafold 3 prediction of Goslar gp058, with secondary structure elements corresponding to the core PF12699 fold shown in red.

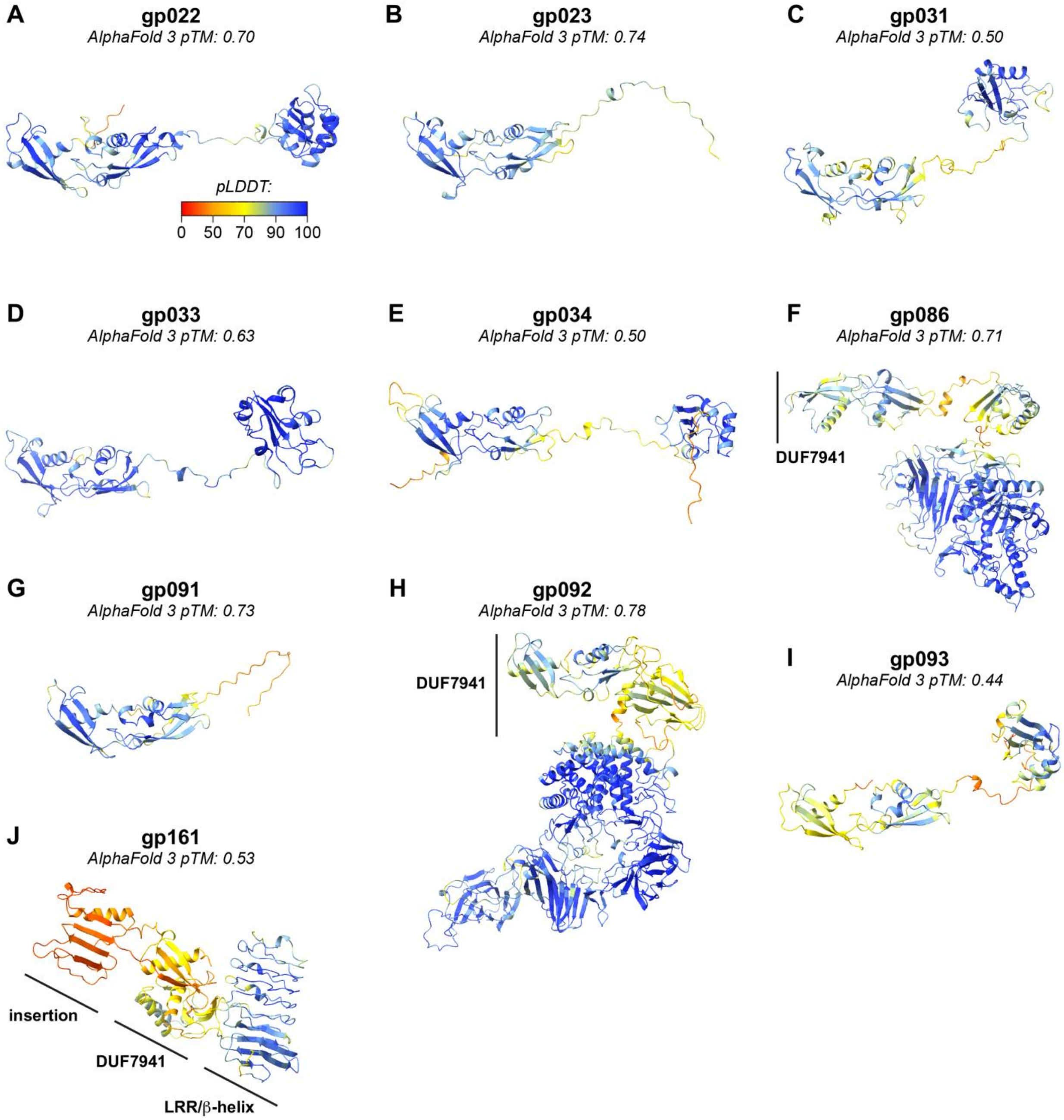

Figure S18. Predicted structures of Goslar DUF7941 proteins. (A) AlphaFold 3 prediction of Goslar gp22, colored by local confidence (pLDDT). **(B)** AlphaFold 3 prediction of Goslar gp23. **(C)** AlphaFold 3 prediction of Goslar gp31. **(D)** AlphaFold 3 prediction of Goslar gp33. **(E)** AlphaFold 3 prediction of Goslar gp34. **(F)** AlphaFold 3 prediction of Goslar gp86. **(G)** AlphaFold 3 prediction of Goslar gp91. **(H)** AlphaFold 3 prediction of Goslar gp92. **(I)** AlphaFold 3 prediction of Goslar gp93. **(J)** AlphaFold 3 prediction of Goslar gp161.

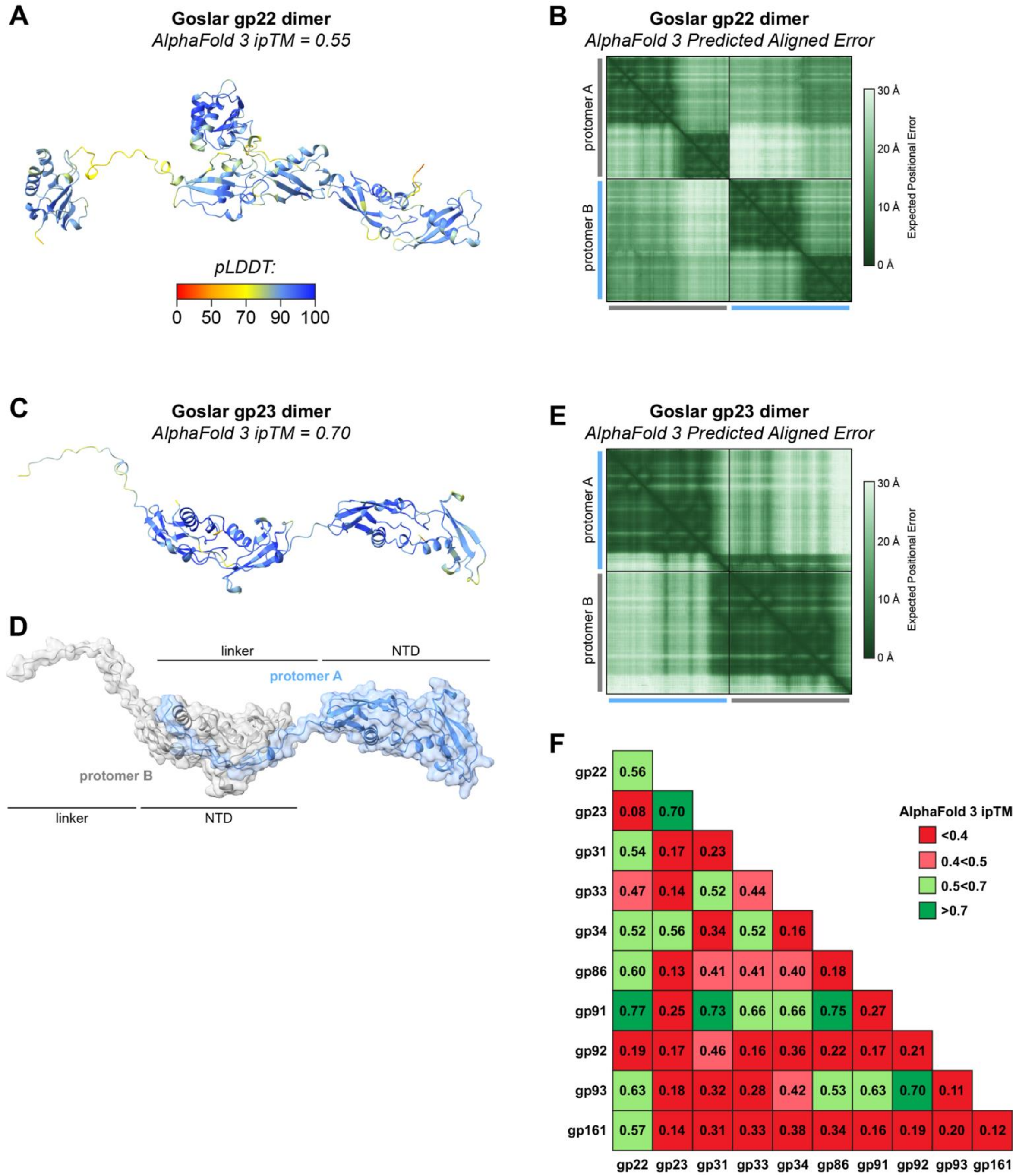

Figure S19. Oligomerization of DUF7941 proteins. (A) AlphaFold 3 prediction of a Goslar gp22 dimer, colored by local confidence (pLDDT). View is equivalent to **Figure 8B**. **(B)** AlphaFold 3 predicted aligned error (PAE) plot for the prediction of a Goslar gp22 dimer. **(C)** AlphaFold 3 prediction of a Goslar gp23 dimer, colored by local confidence (pLDDT). **(D)** AlphaFold 3 prediction of a Goslar gp23 dimer, colored by protomer (blue/gray) and with DUF7941 subdomains of each protomer indicated. **(E)** AlphaFold 3 predicted aligned error (PAE) plot for the prediction of a Goslar gp23 dimer. **(F)** AlphaFold 3 ipTM values for pairs of Goslar DUF7941 proteins. Shades of red indicate low confidence of interaction, and shades of red indicate increasing confidence of interaction.

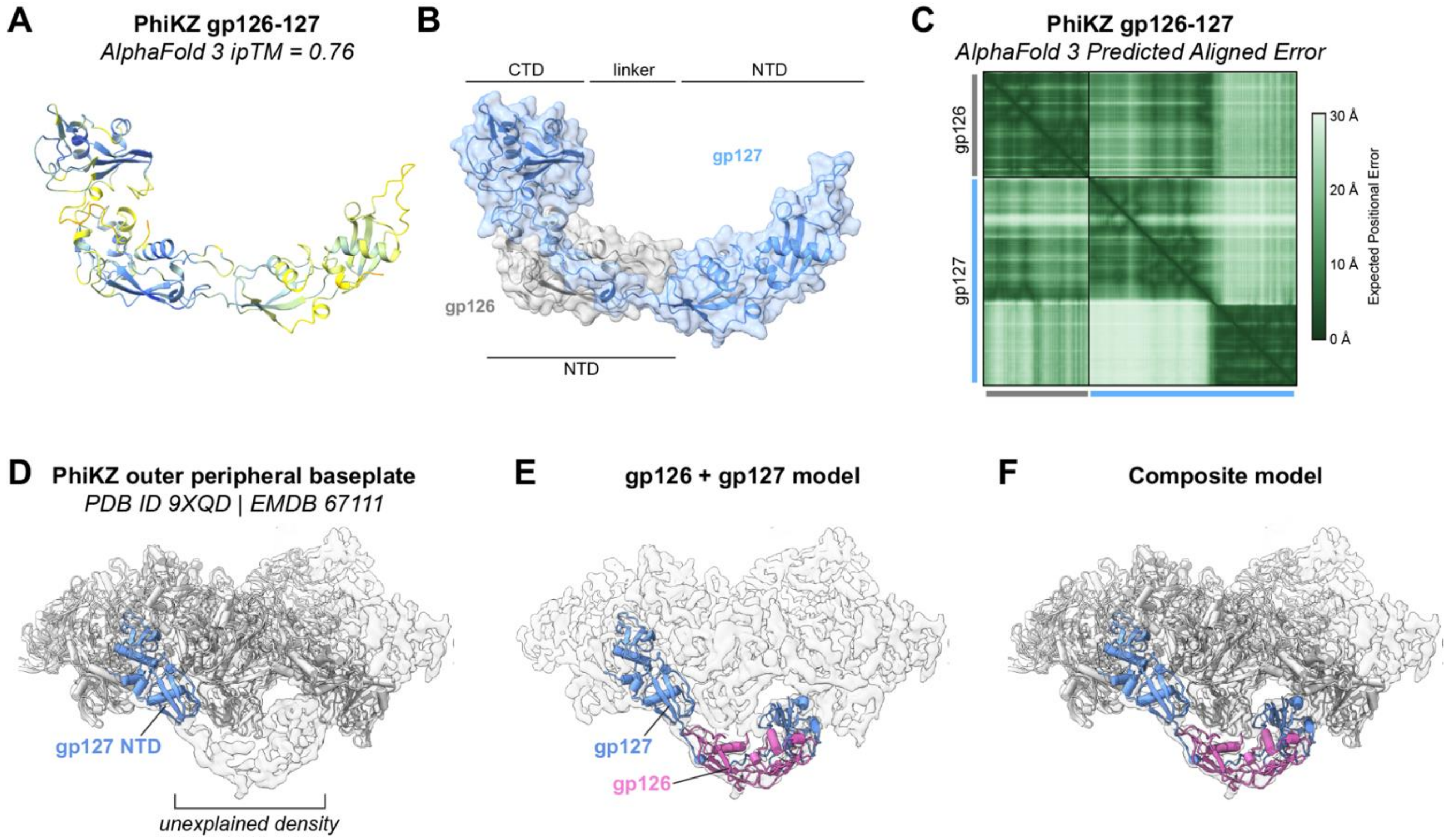

Fig. S20. PhiKZ gp126-gp127 structure. (A) AlphaFold 3 prediction of a PhiKZ gp126-gp127 heterodimer, colored by local confidence (pLDDT). **(B)** AlphaFold 3 prediction of a PhiKZ gp126-gp127 heterodimer, colored by protein (gp127 blue, gp126 gray) and with DUF7941 subdomains of each protein indicated. **(C)** AlphaFold 3 predicted aligned error (PAE) plot for the prediction of a PhiKZ gp126-gp127 heterodimer. **(D)** PhiKZ outer peripheral baseplate structure (PDB ID 9XQD; EMDB-67111) *(34)*, with gp127 (NTD; residues 1-150) shown in blue. Unexplained density adjacent to the gp127 NTD model is noted. **(E)** AlphaFold 3 model of the PhiKZ gp126-gp127 heterodimer manually fit into the outer peripheral baseplate map. Gp126 (pink) and the linker+CTD of gp127 (residues 151-290) fit into unexplained density. Fitting involved (1) overlaying the heterodimer model on the already-built gp127 NTD, (2) splitting the model between gp127 residues 150 and 151, then (3) rotating the (gp126 plus gp127 linker+CTD) coordinates ~20° to fit the unexplained density. **(F)** Composite model showing the PhiKZ outer peripheral baseplate model (gray) plus the gp126-gp127 heterodimer.

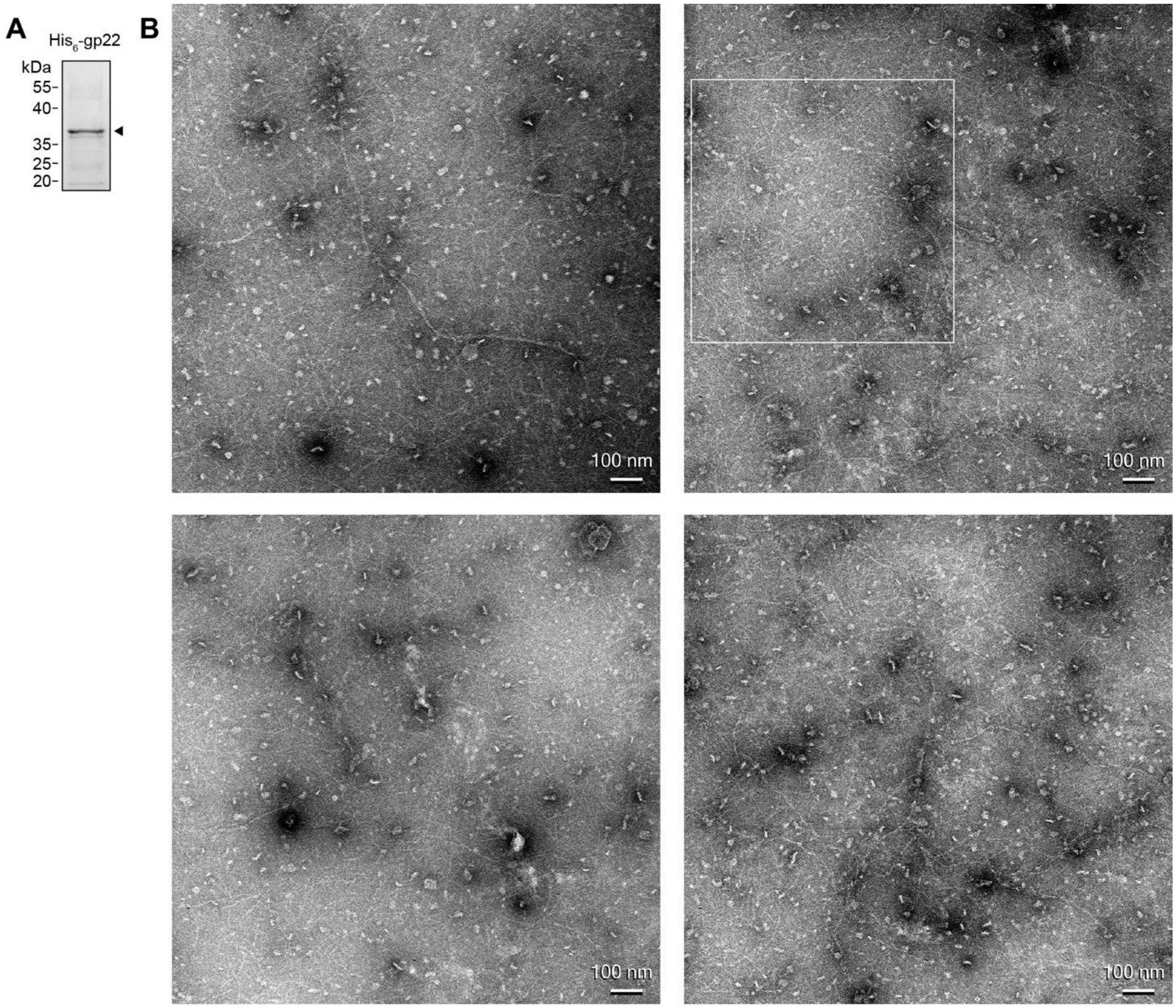

Fig. S21. Negative stain-electron microscopy of Goslar gp22. (A) SDS-PAGE analysis of Goslar His$_6$-gp22 after Ni^{2+} affinity purification in mildly denaturing conditions (2 M urea). The A_{260}/A_{280} ratio of the purified protein was 0.6, indicating that there was not significant nucleic acid contamination. **(B)** Representative negative-stain electron microscopy images of purified Goslar His$_6$-gp22 after exchange to a buffer without urea, showing filament formation. The white box on the upper-right image indicates the area shown in **Fig. 8C**. Scale bar = 100 nm.

Table S1. Goslar structural proteins

Goslar protein	NCBI ID	Common name	Copy number	PhiKZ homolog
HOV27_gp041	YP_009820726	Major capsid protein	1615	PHIKZ120
HOV27_gp146	YP_009820831	Capsid latch	55	PHIKZ244
HOV27_gp077	YP_009820762	Capsid decorator	60+	PHIKZ081
HOV27_gp042	YP_009820727	Vertex binding complex	55	PHIKZ119
HOV27_gp059	YP_009820744	Vertex binding complex	55	PHIKZ091
HOV27_gp065	YP_009820750	Vertex binding complex	55	PHIKZ086
HOV27_gp066	YP_009820751	Vertex binding complex	110	PHIKZ085
HOV27_gp192	YP_009820927	Vertex binding complex	55	PHIKZ153
HOV27_gp036	YP_009820721	Portal	12	PHIKZ129
HOV27_gp233	YP_009820886	Adaptor	12	PHIKZ042
HOV27_gp050	YP_009820735	Stopper	12	PHIKZ099
HOV27_gp051	YP_009820736	Tail terminator	6	PHIKZ098
HOV27_gp126	YP_009820811	Collar	60+	N/A
HOV27_gp217	YP_009820902	Tail tube	276	PHIKZ030
HOV27_gp218	YP_009820901	Tail sheath	282	PHIKZ029
HOV27_gp172	YP_009820857	Baseplate upper wedge	6	N/A
HOV27_gp063	YP_009820748	Baseplate upper wedge	12	PHIKZ087
HOV27_gp035	YP_009820720	Baseplate upper wedge	6	PHIKZ128
HOV27_gp180	YP_009820865	Baseplate upper wedge	6	PHIKZ062
HOV27_gp243	YP_009820876	Baseplate lower wedge	24	PHIKZ049
HOV27_gp187	YP_009820932	Baseplate lower wedge	12	PHIKZ161
HOV27_gp202	YP_009820917	Baseplate lower wedge	6	PHIKZ174
HOV27_gp032	YP_009820717	Inner-peripheral baseplate spokes	60	PHIKZ139
HOZ27_gp220	YP_009820899	Inner-peripheral baseplate spokes	12 (AF3 model)[1]	PHIKZ027
HOV27_gp221	YP_009820898	Peripheral baseplate protein	(not modeled)[2]	PHIKZ026
HOV27_gp022<br>HOV27_gp023<br>HOV27_gp031<br>HOV27_gp033<br>HOV27_gp034	YP_009820707<br>YP_009820708<br>YP_009820716<br>YP_009820717<br>YP_009820718	Peripheral baseplate protein (DUF7941)	(not modeled)	PHIKZ126<br>PHIKZ127
HOV27_gp030	YP_009820715	Peripheral baseplate protein	(not modeled)	PHIKZ130
HOV27_gp029	YP_009820714	Peripheral baseplate protein	(not modeled)	PHIKZ131
HOV27_gp025	YP_009820710	Peripheral baseplate protein	(not modeled)	PHIKZ133-135
HPV27_gp188	YP_009820931	Peripheral baseplate protein	(not modeled)	PHIKZ157
HOV27_gp062	YP_009820747	Tail tube initiator complex	6	PHIKZ088
HOV27_gp203	YP_009820916	Tail tube initiator complex	6	PHIKZ182
HOV27_gp048	YP_009820733	Hub	3 (AF3 model)	PHIKZ101
HOV27_gp013	YP_009820698	Tail spike	3 (AF3 model)	PHIKZ164

[1]Proteins labeled "AF3 model" were not manually built and subjected to atomic refinement; AlphaFold 3 models were docked and fit into density using the ChimeraX "fit in map" command.

[2]Proteins labeled "not modeled" are observed the peripheral baseplate structure of the related phage PhiKZ, but could not be modeled in the peripheral baseplate of Goslar because of limited map quality.

Data S1. (separate file)

Microsoft Excel workbook with cryoEM data collection and structure determination statistics for intact Goslar virions.

Data S2. (separate file)

Microsoft Excel workbook with cryoEM data collection and structure determination statistics for Goslar substructures.

Data S3. (separate file)

Microsoft Excel workbook with LFQ mass spectrometry of purified Goslar virions.
